# Review: Oncolytic virotherapy, updates and future directions

**DOI:** 10.18632/oncotarget.18309

**Published:** 2017-05-31

**Authors:** Christos Fountzilas, Sukeshi Patel, Devalingam Mahalingam

**Affiliations:** ^1^ The University of Texas Health Science Center at San Antonio, San Antonio, TX, USA

**Keywords:** oncolytic virus, immunotherapy, cytotoxicity

## Abstract

Oncolytic viruses (OVs) are viral strains that can infect and kill malignant cells while spare their normal counterparts. OVs can access cells through binding to receptors on their surface or through fusion with the plasma membrane and establish a lytic cycle in tumors, while leaving normal tissue essentially unharmed. Multiple viruses have been investigated in humans for the past century. IMLYGIC™ (T-VEC/Talimogene Laherparepvec), a genetically engineered Herpes Simplex Virus, is the first OV approved for use in the United States and the European Union for patients with locally advanced or non-resectable melanoma.

Although OVs have a favorable toxicity profile and are impressively active anticancer agents *in vitro* and *in vivo* the majority of OVs have limited clinical efficacy as a single agent. While a virus-induced antitumor immune response can enhance oncolysis, when OVs are used systemically, the antiviral immune response can prevent the virus reaching the tumor tissue and having a therapeutic effect. Intratumoral administration can provide direct access to tumor tissue and be beneficial in reducing side effects.

Immune checkpoint stimulation in tumor tissue has been noted after OV therapy and can be a natural response to viral-induced oncolysis. Also for immune checkpoint inhibition to be effective in treating cancer, an immune response to tumor neoantigens and an inflamed tumor microenvironment are required, both of which treatment with an OV may provide. Therefore, direct and indirect mechanisms of tumor killing provide rationale for clinical trials investigating the combination of OVs other forms of cancer therapy, including immune checkpoint inhibition.

## INTRODUCTION

Oncolytic viruses (OVs) are viral strains that can infect and kill malignant cells (oncolysis) while sparing their normal counterparts. Oncolysis can be either a natural property of the virus [naturally occurring OVs, e.g. reovirus] or a consequence of manipulation of the viral genome [genetically engineered OVs, e.g. adenovirus] and renders oncolytic virotherapy – the use of replication-competent virus for cancer treatment – a potential therapeutic modality in cancer treatment.

Oncolytic virotherapy has been studied for the last century. One of the first case reports of a dramatic regression of cervical cancer in a patient receiving the Pasteur-Roux live attenuated rabies vaccine was presented in 1910 [1]. In the 1940s, human studies with different types of viruses were launched [2]. The era of modern oncolytic virotherapy started in the early 1990s, when a genetically modified, live-attenuated, thymidine kinase (TK)-negative herpes simplex virus (HSV) strain was locally injected into human glioma xenograft models, showing promising results [3]. Today, IMLYGIC™ (T-VEC/Talimogene Laherparepvec), a genetically engineered HSV, is the first OV approved for use in the United States and the European Union for patients with locally advanced or non-resectable melanoma [4, 5].

In this review paper, we will discuss the general mechanisms of antitumor activity of OVs, limitations of oncolytic virotherapy, recent advances in clinical development for individual agents, and future prospects.

### General mechanisms of oncolysis

OVs have been generally categorized into DNA and RNA viruses and further divided into double- and single-stranded. OVs can access cells through binding to receptors in their surface or through fusion with the plasma membrane. An essential characteristic of an OV is the ability to establish lytic cycle in malignant but not normal tissues, either by naturally exploiting inherent tumor weaknesses, such as RAS pathway activation [6–8] or by genetic modification. For example, knockdown of TK gene in HSV can lead to preferential killing of tumor cells, as TK-negative HSV can replicate only in dividing cells depending on their TK activity [3, 9, 10]. TK, an important enzyme involved in viral DNA synthesis and repair [11], is highly expressed in activated cells in G1 phase *in vitro* [12]. OVs have the ability to establish a niche of continuous viral replication within the tumor, recruit uninfected cells in proximity creating syncytia, infect dividing and non-dividing cells, and be stable *in vivo*, yet lack chromosomal integration and do not result in major disease [13]. OVs, like reovirus [14], HSV [15, 16] or vaccinia virus [17], can induce tumor-specific adaptive immune responses and indirectly cause malignant cell death. Adenovirus [18], Coxsackie B3 [19] and measles virus [20] can lead to endoplasmic reticulum (ER) stress and cause immunologic cell death – a type of cell death that leads to release of danger-association molecular patterns, like adenosine triphosphate, calreticulin and high-mobility group box-1, which attract immune cells [21].

OVs can also selectively target tumor neo-vasculature. Vesicular stomatitis virus (VSV) can selectively infect endothelial cells in the tumor microenvironment and cause thrombosis in the tumor vessels [22]. HSV and vaccinia virus can also selectively damage tumor endothelium [23, 24]; preferential replication in tumor vessels may be secondary to the dependence on high vascular endothelial growth factor (VEGF) and fibroblast growth factor (FGF) levels for replication in normal endothelium [24]. OVs can be genetically engineered to express anti-angiogenic factors, like VEGF inhibitors [25, 26]. Vaccinia virus treatment can lead to a decrease in perfusion within the tumor and suppression of VEGF levels, which are restored after viral clearance, resulting in a synergistic antitumor activity with VEGF receptor (VEGFR) tyrosine kinase inhibitors (TKIs) [27]. Synergy with VEGFR TKIs may be a result of off-target inhibition of cellular antiviral defense proteins, like the double stranded RNA-dependent protein kinase (PKR) [28, 29].

### Limitations of oncolytic virotherapy

OVs are not the ‘magic bullet’ for cancer therapy. Single agent activity is modest for most agents. The main limitation of oncolytic virotherapy is accurate delivery to the target. The ideal method of delivery is systemic, preferably intravenous (IV). In order for an OV to establish a niche within the tumor after systemic administration, the OV has to bypass the liver that may actively sequester a percentage of the administered dose [30]. Administering the virus directly within the tumor overcomes this limitation, mainly in the minority of tumors with easily accessible skin and subcutaneous lesions such as melanoma, with an abscopal effect and dissemination in distant sites [31–34]. For the majority of disseminated malignancies with visceral or osseous metastasis, the logistics of intralesional administration may prohibit its use. Further, as most OVs are ubiquitously present in nature and humans are infected at an early age or vaccinated against some, most patients have neutralizing antibodies that can bind the virus and limit target delivery [35]. For example, 50–80% of humans possess neutralizing antibodies against HSV and almost 90% against reovirus [36, 37]. Finally, the adaptive immune system may be a double edge sword, playing a role in both tumor killing and early elimination of viral infection through humoral (e.g. antibody and complement binding) and cellular mechanisms [38]. Preclinical data can often be misleading, as in immunocompromised mouse xenograft models an immune response cannot be generated leading to under- or overestimation of the agent efficacy. Using syngeneic immunocompetent models has been proposed as a possible solution to this problem, but the agent activity in a human vs. animal cell may be different.

**Figure 1:**
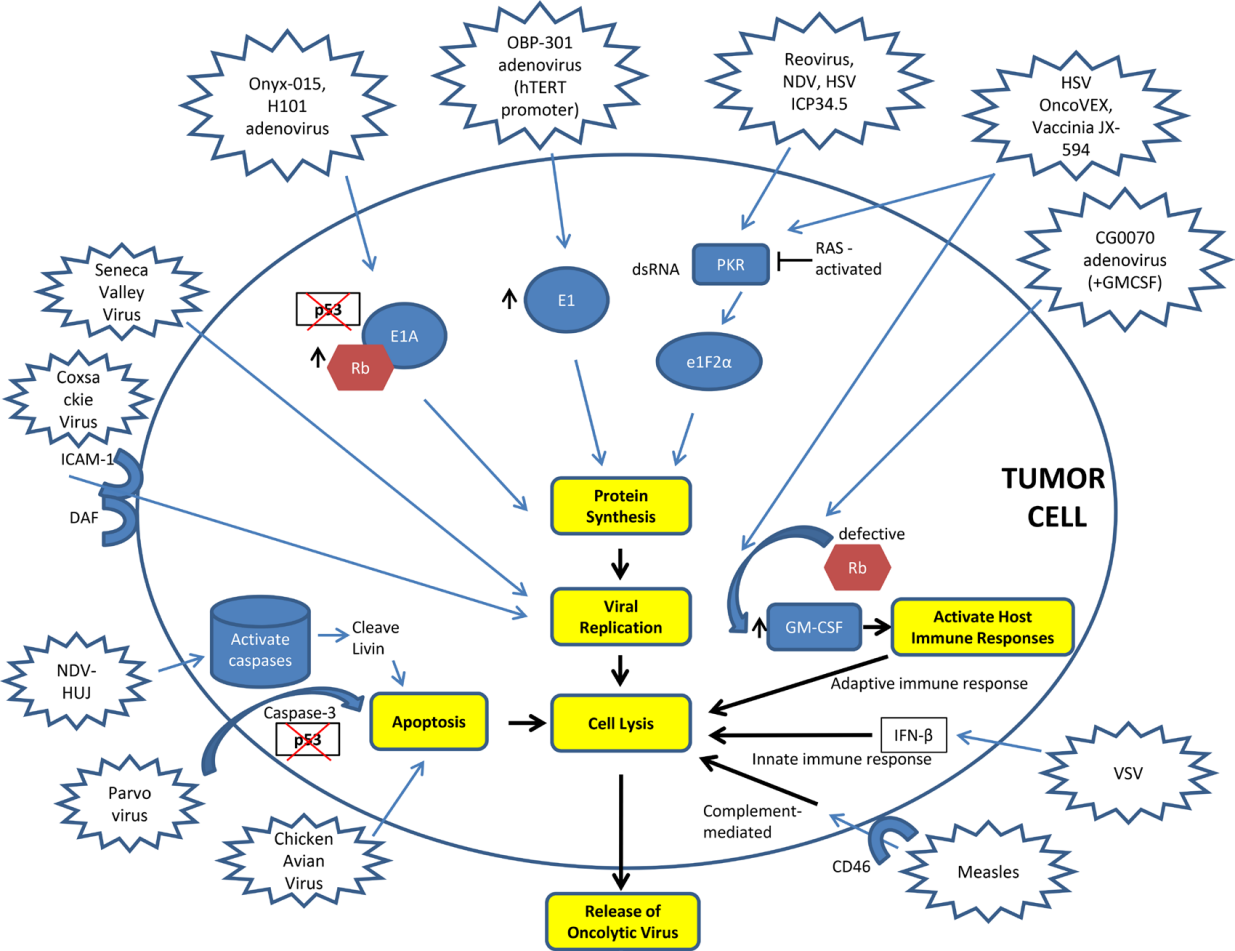
Mechanisms of action of oncolytic viruses DAF – Decay Accelerating Factor, GM-CSF – Granulocyte Macrophage-Colony Stimulating Factor, HSV – Herpes Simplex Virus, hTERT – Human Telomerase, ICAM-1 – Intercellular Adhesion Molecule-1, ICP – Infectious Cell Protein, INF-β – Interferon beta, NDV – Newcastle Disease Virus, VSV – Vesicular Stomatitis Virus.

Direct and indirect mechanisms of tumor killing provide rationale for the combination of OVs with cytotoxic, anti-angiogenic and immune therapies in patients with cancer. We will discuss individual agents in subsequent sections. Tables 1A, 1B and 2A, 2B and Figure 1 summarize individual agent design, mechanisms of action, preclinical and clinical data.

**Table 1A:** Summary of DNA, double-strand virus design, mechanisms of action and clinical implications

Virus	Design	Clinical Implications
Herpes Simplex Virus (HSV)	· T-VEC: ICP34.5/ICP47 mutant and US11/GM-CSF expressing [31]	· Melanoma [32, 33].
	· G207: ICP34.5/RR mutant [43, 61–63]	· Glioma [68, 69].
	· NV1020: ICP34.5 mutant [70, 71].	· CRC [72, 73].
	· HFA10: RR mutant [75]	· Data for breast, head and neck, and pancreas cancer [74–76]
Vaccinia Virus	· vvDD: TK mutant strain. Viral protein VGF binding to EGFR in cell surface [185–187].	· Phase I: ITu administration safe; abscopal effect [188].
	· JX-594: TK mutant / GM-CSF expressing strain [189].	· HCC [84] · Other solid tumors including CRC [85, 86]
	· GL-ONC1: TK mutant / HA expressing [190].	· Malignant Effusions: Intrapleural injection safe; prolongs disease stability in malignant mesothelioma [191]. · Peritoneal Carcinomatosis: IP administration safe [192]. · Solid tumors: IV administration, 18% SD as best response (lasting >6 months) – IHC revealed delivery to the tumor [193, 194].
Adenovirus	· ONYX-015: E1B55 mutant [90]	· Head and Neck [92].
	· Adenovirus chimeras · Ad5-D24: serogroup 5, E1A-/Rb-binding site negative; selectively kills cells with an abnormal p16/Rb pathway [195] · CRAd: Ad5-D24 plus serogroup 3 knob [196] · DNX-2401: Ad5-D24 plus RDG (integrin receptor) [197] · Ad5/3-D24-GMCSF and CGTG-102: CRAd and DNX-2401 plus GM-CSF respectively [197, 198]. · ColoAd1: tumor selective Adenovirus 11/3 · Ad5/3, hTERT and CD40 ligand expressing strain has improved antitumor immunity/activity [18].	· Phase I: CRAd by IP safe, 60% SD [196]. · Phase I: DNX-2401 by ITu administration in glioma safe, 12% CR rate, 11 months OS [199] · Phase I: DX-2401 or Ad5-D24-RDG-GMCSF ITu in solid tumors safe, 27% with SD [197]. · Phase I: CGTG-102 ITu in sarcoma safe, 75% with SD [198]. CGTG-102 ITu in solid tumors plus oral low-dose cyclophosphamide safe, 40% with controlled disease at 3 months [172]. · ColoAd1 has low level of pre-existing host immunity [200] and potentially higher potency than ONYX-015 [201]. It is tested in a mechanism of action Phase I study [202].
	· CG0070: E1A gene is under the control of E2F, GM-CSF expressing [94].	· Urothelial carcinoma [96]
	· CV764 and CN706: E1A gene is under the regulation of PSA [203, 204].	· Potential role in prostate cancer
	· OBP-301: hTERT promoter regulates the expression of E1 genes [205].	· Preclinical activity in a CRC [206]

CR: complete response, CRC: colorectal cancer, DNA: deoxyribonucleotide nucleic acid, EGFR: epidermal growth factor receptor, GM-CSF: granulocyte macrophage-colony stimulating factor, HA: hemagglutinin, HCC: hepatocellular carcinoma, hTERT: human telomerase, ICP: infectious cell protein, IHC: immunohistochemistry, IP: intraperitoneal, ITu: intratumoral, IV: intravenous, RR: ribonucleotide reductase, SD: stable disease, TK: thymidine kinase, US11: HSV RNA binding protein, VGF: viral growth factor.

**Table 1B:** DNA, single-strand viruses design, mechanisms of action and clinical implications

Virus	Design	Clinical Implications
Parvovirus	· H-1PV: Selectivity for mutated or inactivated p53; transformed cells are more vulnerable to H-1PV than normal cells [101]. · H-1PV-infected melanoma cells can activate dendritic cells as well as cross-prime cytotoxic T-cells [207].	· Preclinical data in glioma: selective replication, prolonged remissions and increased survival observed with ITu+IV H-1PV [208]. · Can cause direct and indirect lysis of tumor cells through stimulation of the immune system against uninfected tumor cells [209]. · Clinical data in glioma [102, 103].
Chicken Anemia Virus	· Induction of apoptosis through viral proteins 2 and 3 [210]; viral protein 3 (Apoptin), causes p53-independent apoptosis specifically in tumor cells [211]. · Bcl-2 protein can stimulate its apoptotic activity [212–214].	· Preclinical data in HCC: systemic delivery can induce apoptosis [215]. · Viral protein 3 combined with chemotherapy *in vitro*, demonstrated increased cytotoxicity [216]; *in vivo* induced apoptosis and caused tumor regression after ITu delivery into Rous sarcoma virus-induced tumors [217]. · No clinical studies reported

DNA: deoxyribonucleotide nucleic acid, HCC: hepatocellular carcinoma, ITu: intratumoral, IV: intravenous.

### DNA viruses

#### Herpes simplex virus (HSV)

T-VEC is a genetically engineered oncolytic HSV, with mutations in infectious cell proteins (ICP) 34.5 and 47, while expressing US11 and granulocyte macrophage-colony stimulating factor (GM-CSF) [31]. GM-CSF is an immunomodulator that enhances viral oncolysis [39]. ICP34.5 protein is necessary for viral replication, viral exit from cells, avoidance of the early shut-off of protein synthesis, and neurovirulence [40–43]. In eukaryotic cells infected with HSV, PKR is activated and phosphorylates the eukaryotic translation initiation factor 2 (eIF-2) thus terminating protein synthesis; ICP34.5 activates the eIF-2 phosphatase to allow for reactivation of eIF-2 and continuation of protein synthesis [42]. In the absence of efficient ICP34.5 activity, the cellular PKR-induced inhibition of protein synthesis and viral replication cannot be reversed [42, 44]. Cellular growth arrest and DNA damage-inducible protein 34 (GADD34, also known as MyD116), has a carboxy-terminal domain homologous to the ICP34.5 corresponding region and can restore protein synthesis in infected cells [45]; non-functional GADD34 in tumor tissue [46] can be potentially responsible for the tumor selectivity of ICP34.5 mutant strains. ICP34.5-mutant HSV can specifically induce cell death in malignant cells either by enhanced replication or by early shut-off of protein synthesis [47] and is non-neurovirulent [48]. US11, a viral ribosome-associated protein, can decrease the levels of active PKR [49]. ICP47 is an inhibitor of transporter associated with antigen presentation (TAP), a protein responsible for transferring the antigen-major histocompatibility complex (MHC) type I complex to the plasma membrane, leading to impaired antigen presentation in HSV-infected cells [50, 51].

Hu et al. published the results of a Phase I trial of T-VEC administered by intratumoral (ITu) injection in patients with multiple tumor types with accessible lesions [31]. Twenty-six of the 30 patients enrolled were evaluable. The most common toxicities were constitutional and injection-site reactions, with grade 3/4 adverse events (AEs) only in HSV seronegative patients. Disease stabilized in three patients, with no difference based on serologic status. On-treatment biopsies revealed tumor necrosis, extensive in some cases, with no necrosis and very rare presence of HSV antigens by immunohistochemistry in normal tissue present in biopsy specimens.

In a phase II study of T-VEC in 50 patients with non-resectable stage III/IV melanoma with lesions amenable to injection, subjects were initially treated with a total ITu injection of up to 4 × 10^6^ pfu (plaque-forming units) followed in 3 weeks later by injections up to 4 × 10^8^ pfu repeated every 2 weeks until disease progression (maximum of 24 injections) [33]. Up to 10 lesions were treated per visit. The overall response rate (ORR) was 26%; eight patients attained a complete response (CR). Most of the responses were sustained for > 6 months and occurred after the first 2 injections; in 6 patients, transient, local or distant progression preceded the response. Again, the HSV serological status did not appear to affect response rate. Responses in non-injected sites were observed. The disease control rate was 50%, and the 1-year overall survival (OS) was 58% (> 90% with response). AEs were mostly constitutional, with grade 3/4 events infrequent.

This study led to a Phase III, multicenter, randomized-controlled trial comparing T-VEC using the previous dosing schema, to subcutaneous GM-CSF (control arm) in the same patient population (OPTiM) [32]. The primary outcome was durable disease response (DDR-lasting more than 6 months). At the time of study was designed there were no life-prolonging therapies approved for disseminated melanoma and as such using a modestly active agent like GM-CSF (or placebo) as the control arm was reasonable. Four hundred thirty-six patients were randomized, with two-thirds of the patients having stage IV disease and 50% patients having received prior therapy. One-third of the patients were HSV seronegative. This trial reached its primary endpoint with DDR of 16.3% in the T-VEC arm compared with 2.1% in the GM-CSF arm (OR 8.9; *P* < 0.001). DRR was higher in treatment-naïve patients and patients with earlier stage disease; there was no difference based on the patients’ HSV serological status. Median time to response was 4.1 months. The same percentage of patients in both arms went on to receive subsequent therapies. There was a trend for improved OS compared to the control arm (23.3 vs.18.9 months in GM-CSF arm, *P* = 0.051). T-VEC was tolerable.

T-VEC is now an approved therapy for non-resectable melanoma. Combination trials with immunotherapy are ongoing. In an ongoing phase I/II study, the combination of T-VEC and ipilimumab in 18 patients with unresectable melanoma resulted in a DDR of 44%, response rate (RR) by immune-related response criteria was 56% with a third of the patients attaining CR. The immune-related response criteria do account for the potential increase in tumor size and appearance of new lesions secondary to treatment-induced tumor inflammation and is probably a better tool for evaluating responses in trials evaluating agents such as OVs or immune checkpoint inhibitors [52, 53]. Grade 3/4 treatment-related AEs occurred in a third of the patients. Two patients developed grade 3/4 immune-related AEs. The median time to response and progression-free survival (PFS) were 5.3 and 10.6 months, respectively. Twelve-and 18-month OS were 72.2% and 67% [54, 55]. Long et al have reported early outcomes of a combination strategy with pembrolizumab (MASTERKEY-265) [56–58]. Thirty-three percent of the patients experienced grade 3/4 AEs attributed to therapy. One patient developed cytokine release syndrome secondary to study treatment. The RR by immune-related response criteria was 48% with 14% of the patients attaining a CR.

Phase I studies have shown HSV replication after ITu injection into malignant brain tumors is safe and efficacious, and therefore, possibly effective as adjuvant therapy [59, 60]. G207 is an ICP34.5- and ribonucleotide reductase (RR)-mutated HSV [43, 61–63]. RR levels are elevated in dividing tumor tissue while not in normal ones [64–66]. In Phase I studies, G207 was administered by ITu injection into gliomas [67]. No toxicity was observed; radiographic and neuropathologic evidence of anti-tumor activity was suggested [67]. Also, multiple ITu dose delivery of G207 is safe before and after resection of malignant glioma [68] as well as concurrently with radiotherapy for recurrent glioma [69].

NV1020 is another HSV-1 strain, attenuated by ICP34.5 knockdown [70, 71]. NV1020 through hepatic artery infusion has been shown to be feasible, safe and potentially efficacious in the treatment of colorectal cancer liver metastasis [72, 73]. The ITu injection of HF10, a highly attenuated HSV-1, into metastatic head and neck cancer sites and recurrent breast cancer sites has shown to cause tumor cell death without any significant AEs [74, 75]. In another pilot study, six patients with non-resectable pancreatic cancer were treated with HF10 during laparotomy and through a catheter for three subsequent injections [76]. No subsequent treatment was administered in the next 30 days. No AEs were reported and 4 patients had clinical benefit (3 with stable disease (SD), 1 with partial response [76]. In a Phase I study, 24 patients with refractory, superficial cancers were treated with ITu administration of HF10 [77]. The treatment was safe; no efficacy data have been reported to date.

#### Vaccinia virus (VAC)

In early Phase I studies, ITu JX-594, a genetically engineered TK-mutant/GM-CSF expressing VAC, demonstrated highly promising results in patients with melanoma [78] and hepatocellular carcinoma (HCC) [79]. In a proof of principle study in patients with melanoma, 10 patients received ITu injections of JX-594 [80]. VAC replication was noted in non-injected lesions as well as intense perivascular lymphocytic infiltration. In another Phase I trial, direct injection of JX-594 into HCC was well tolerated and associated with viral replication, decreased tumor perfusion, and tumor necrosis [81]. In this study, the safety and efficacy of JX-594 followed by sorafenib was assessed in three patients. The sequential therapy regimen was well tolerated, associated with decreased tumor perfusion, and associated with objective tumor responses (using the Choi criteria [82, 83], a more appropriate response evaluation method for HCC that takes into account both target lesion size as well as perfusion), with tumor necrosis up to 100%. This study concluded that treatment of HCC with JX-594 followed by sorafenib has antitumor activity, and JX-594 may sensitize tumors to subsequent therapy with VEGFR TKIs.

In a randomized Phase II dose finding study, JX-594 was administered by ITu injection in 30 patients with unresectable HCC [84]. One severe treatment-related AE was recorded (nausea and vomiting), and all patients experienced grade 1/2 flu-like illness. One patient developed grade 1 pustular skin rash. The modified Choi RR was 62%, the disease control rate at week 8 was 46% overall (by modified RECIST). Responses were observed in injected as well as non-injected tumors. The OS was 14.1 months for the high-dose group (vs. 6.7 months in the low-dose). Of note, 25% of the patients in the high-dose group had disease refractory to sorafenib, whereas none in the low-dose group.

JX-594 has also demonstrated systemic delivery potential. The results of a Phase I dose-escalation trial of IV JX-594 in patients with metastatic tumors showed selective infectivity, replication, and expression of transgene products in cancer tissue in a dose-related fashion [85]. Normal tissues (adjacent normal tissues excised in on-treatment biopsies as well as blood mononuclear cells) were not affected [85].

JX-594 was tested in a Phase I dose-escalation trial in patients with metastatic, refractory colorectal cancer [86]. Fifteen patients were treated at three different dose levels IV every 2 weeks (total 4 doses). All AEs were grade 1/2 (no dose-limiting toxicity-DLT); most common were fever and chills (90%). One patient in the high dose group developed a pustular rash. Eighty-nine percent of the patients at the highest dose experienced disease stabilization. TRAVERSE, a Phase II study comparing intravenous and intratumoral JX-594 plus best supportive care to best supportive care alone in patients with advanced HCC who have progressed on sorafenib, is underway [87]. A randomized Phase III study of sorafenib vs. sorafenib following JX-594 in sorafenib-naïve patients is open to accrual (NCT02562755).

#### Adenovirus (Ad)

Oncolytic Adenoviruses are been genetically modified to take advantage of the altered tumor environment [88, 89]. ONYX-015 is an E1B55-mutant Ad that can cause oncolysis of cancer cells with mutant p53 [90]; although it has been shown to replicate independent of p53 [91]. In a phase II study of 37 patients with head and neck cancer, ONYX-015 by ITu injection was combined with chemotherapy. In this non-randomized trial, investigators compared RR and time to progression in injected vs. non-injected tumors with superior outcomes in injected tumors [92]. ONYX-015 was approved for human use in China in 2006, [93] but its clinical development in the United States and Europe has been halted since 2000 due to lack of efficacy.

CG0070 is a GM-CSF expressing Ad where the E1A gene is under the control of E2F, a retinoblastoma (Rb)-dependent transcription factor, providing selectivity for cells with abnormal p16/Rb pathway [94]. When Rb binding to E2F is lost, E2F remains transcriptionally active [95]. CG0070 has selective replication, cytotoxicity, production of GM-CSF and antitumor efficacy in urothelial carcinoma [94]. A first-in human Phase I trial of intravesical administration of CG0070 included 35 patients with non-muscle invasive urothelial cancer progressing after bacille Calmette-Guerin (BCG) therapy (4 different dose levels, single or multiple doses) [96]. Therapy was well tolerated with mostly grade 1 or 2 bladder AEs. Increased GM-CSF in urine and viral replication were observed. The RR was 48.6% and the median duration of response was 10.4 months. Increased expression of Rb by IHC was associated with higher RR (58 vs. 20%); however, the cohort of patients was too small for definitive conclusions.

#### Parvovirus (PV)

PV B19 can induce cell death through apoptosis in erythroid cells through non-structural proteins (NS1)-induced caspase-3 activation [97–100]. PV has selectivity for mutated or inactivated p53; transformed cells are more vulnerable to H-1PV than normal cells [101]. ParvOryx01 is a first in human, Phase I/IIa, dose-escalation study of H-1PV given locally and systemically in patients with recurrent glioma. The first part of the study (treatment of nine patients with ITu injection followed by tumor resection and virus injection in the tumor margin) has been completed without DLT [102]. Correlative studies in resected tumors showed the ability of H-1PV to cross the blood-brain barrier, spread and replicate in tumor tissue [103]. There was also evidence of induction of anti-tumor immunity, though clinical efficacy outcomes have not been reported.

### RNA viruses

#### Reovirus (RV)

Reovirus preferentially targets cancer cells based on their higher rates of cell division, which differs from that of normal cells, reviewed by Gong and Mita [104]. An unmodified, nonpathogenic, type 3 Dearing reovirus strain (Reolysin) has been extensively evaluated in preclinical models and clinical studies. This RV has a dual mechanism of action including the selective lysis of tumor cells and induction of an anti-tumor immunity. The selective permissiveness of cancer cells to reovirus replication and lysis, not observed in normal cells, is dependent on a number of factors both, endogenous and exogenous. The former include: 1) defective PKR signaling; 2) RAS activation and/or mutations in upstream and downstream RAS-effector proteins that downregulate the IFN-induced antiviral response and 3) dysfunctional or deleted tumor suppressor-genes (e.g., p53 and ATM) [8, 104–106]. Exogenous factors include cellular stress resulting from chemo- and/or radiotherapy and reovirus modulation of interferon signaling [14, 107]. One or more of these factors allows for viral translation, replication, oncolysis and cancer cell death [8]. The presence of “infected” tumor cells and the release of viral- and tumor-associated antigens after tumor cell lysis, induce robust innate and adaptive antitumor immune responses [14, 108–110]. Therapy using a wild-type RV type 3 Dearing (Reolysin), has also demonstrated synergy and/or additive effects with standard chemotherapies [107, 111–114] and immunosuppressant agents [115]. Combination therapy can overcome pre-existing immunity to RV without affecting metastatic tumor regression. RV has been shown to preferentially infect, induce ER stress and kill RAS-activated pancreatic cancer cells [116, 117]. Furthermore, IP administration of RV inhibits the peritoneal dissemination of pancreatic cancer cells in a syngeneic immunocompetent animal model [118]. Intraportal administration of RV has decreased the number and size of treated tumors in the same model [119].

Reolysin is one of the best-studied OV and several Phase I and II studies have been completed. Over 1,000 patients have received single or multiple doses, IV or ITu, either as monotherapy or in combination with radiotherapy or chemotherapy (Table 2A). The most common AE related to Reolysin is a flu-like illness. It does not appear to increase toxicities in combinations with cytotoxic agents.

**Table 2A:** RNA, double-strand virus design, mechanisms of action and clinical implications

Virus	Design	Clinical Implications
Reovirus	· RAS-induced inhibition of cellular PKR is responsible for preferential activity in RAS-activated cells, allowing viral translation, replication, oncolysis and cancer cell death [8]. · RAS activated JNK and NFkB can mediate reovirus- induced apoptosis [218, 219]. · Reovirus can induce antitumor immune responses [14]. · Reovirus can preferentially infect and kill pancreatic cancer cells [116, 117]	· Preclinical data: wild-type reovirus type 3 Dearing (Reolysin), has demonstrated synergy and/or additive effects with standard chemotherapies [107, 111–114]. · Preclinical data: the combination with immune-modulating chemotherapeutic drugs may enhance the antitumor effects by attenuating the antibody response, allowing enhanced viral replication and circulation for longer time periods, and enhancing the antitumor immune effect [168, 170]. Combination therapy with immunosuppressant agents has synergistic antitumor activity and appeared to overcome a pre-existing immunity without affecting metastatic tumor regression [115]. · Preclinical data in pancreas cancer: inhibition of the peritoneal dissemination of pancreatic cancer cells [118]; activity with intraportal administration [119]. · Phase I: IV Reolysin established as a safe therapy [220, 221]. · Phase I: ITu Reolysin in glioma [222] and advanced superficial malignancies safe [223]. · Phase I: Reolysin infused locally for 72 hours in patients with gliomas safe, 66% SD – one PR [224]. · Combinations with docetaxel [225], paclitaxel/ carboplatin [226] and gemcitabine [171] are well tolerated. · Clinical data for NSCLC, CRC, breast and pancreas cancers [120–122, 124, 227].

CRC: colorectal cancer, ITu: intratumoral, IV: intravenous, JNK: c-Jun N-terminal kinase, NFkB: nuclear transcription factor kappa B, NSCLC: non-small cell lung cancer, PKR: double-stranded RNA-dependent protein kinase, RNA: ribonucleotide nucleic acid, PR: partial response, SD: stable disease.

**Table 2B:** RNA, single-strand virus design, mechanisms of action and clinical implications

Virus	Design	Clinical Implications
Coxsackie Virus	· Coxsackie A21 (CVA21): ICAM-1 and DAF forms the cellular receptor for CAV21 resulting in specific viral attachment, cell internalization, and subsequent rapid cell lysis [129], DAF is upregulated on surfaces of many tumors [127, 128]. · Coxsackie B3 (CVB3): The receptors for CVB3 are DAF and CAR. Normal lung cell lines express low levels of CAR but moderate to high for NSCLC cells [19].	· Preclinical data in Melanoma: activity with ITu, IP or IV CVA21 [130]. · Preclinical data in breast cancer: IV CVA21 plus doxorubicin had synergistic effects [228]. · Preclinical data for NSCLC: CVB3 was found to be active and selective [19]; abscopal effect was noted. · Clinical data in melanoma for CVA21 [131].
Measles Virus (MV)	· MV vaccine affects only cells with a high density of CD46, and therefore, does not affect normal cells [137]. · MV-GFP-H(AA)-IL-13: human IL-13 displayed at the C-terminus of the H protein; CD46 and SLAM-ablating mutations in H protein. Intracerebral administration has shown efficacy as well as lack of neurotoxicity [229]. · MV-CEA: expresses the CEA gene [230] · MV-NIS: expresses NIS; distribution can be monitored by functional imaging [231, 232] and allows intracellular transfer of ^131^I and potentiation of antitumor activity.	· Preclinical studies in solid tumors and hematologic malignancies have evaluated measles virus through different routes (ITu, IV, IP or intrapleural) and administration schedules [140–144]. Treated animal tumors demonstrate cytopathic effect with syncytia formation followed by apoptotic cell death of MV-infected tumor cells [145]. · No toxicity was observed in clinical or laboratory tests after IP, ITu or IV administration [229, 233–235].
Newcastle Disease Virus (NDV)	· NDV binds cells via HN and fuses using the F protein [151] · PV701 and MTH68/H are live attenuated oncolytic viral strains of NDV, which have the capacity to selectively replicate in and lyse tumor cells, and to be immunostimulatory [152] · NDV-HUJ: can overcome the anti-apoptotic effect of anti-apoptotic protein Livin [156]. · The ER stress is a key component of induction of antitumor immunity [236–238].	· Preclinical data show induction of apoptosis in different cell types [239–241] · Oncolytic strains given via IV, IP, and ITu routes, have been shown to have tumor selectivity [242].
Vesicular Stomatitis Virus (VSV)	· VSV-hINFb: express IFN-β, resulting in effective oncolytic activity with increased antitumor immune response [163, 164]. · Mutation of the matrix gene: unopposed mRNA export from the nucleus, resulting in the stimulation of robust innate immune responses and induces anticancer cytokines. [165, 166]	· A toxicology study in rats and rhesus macaques has shown that ITu injection of oncolytic VSV expressing human interferon-β (VSV-hIFNβ) did not have any observed AEs [167].
Seneca Valley Virus	· The selective tropism of virus replication may involve receptor-mediated internalization [158–160].	· Preclinical data in SCLC: complete and durable responses with systemic administration [161]; preexisting antibodies are rare. SVV-001 can readily penetrate solid tumors from the vascular system. · Solid tumors with neuroendocrine features [162].
ECHO	· ECHO and Coxsackie Virus share mechanisms of cytotoxicity. · ECHO-1: enter cells through binding to the integrin alpha 2 beta 1 [243, 244].	· Preclinical data in ovarian cancer cells [244] and ovariand and prostate cancer xenografts [245]. · In a retrospective, non-randomized comparison, ECHO-7 Rigvir by regional intramuscular administration of Rigvir for up to 3 years in patients with excised stage II, seemed to improve OS even though did not increase disease-free survival [246]. · Rigvir has been approved for treatment of melanoma in Latvia since 2009.

CAR: coxsackie and adenovirus receptor, CEA: carcinomebryonic antigen, CRC: colorectal cancer, DAF: decay accelerating factor, ER: endoplasmic reticulum, HA: hemagglutinin, HN: hemagglutinin neuraminidase , ICAM-1: intercellular adhesion molecule-1, INF-b: interferon beta, IP: intraperitoneal, ITu: intratumoral, IV: intravenous, NIS: sodium iodide symporter, NSCLC: non-small cell lung cancer, OS: overall survival, RNA: ribonucleotide nucleic acid, SCLC: small cell lung cancer, SLAM: signaling lymphocyte–activating molecule.

The Canadian Cancer Trials Group (CCTG) presented positive overall survival (OS) data from an open-label, randomized, phase 2 study assessing the therapeutic combination of intravenously-administered REOLYSIN given in combination with the chemotherapy agent paclitaxel versus paclitaxel alone, in patients with advanced or metastatic breast cancer (IND 213). The 74-patient study, powered to 90% and designed by the CCTG, reported that in the intention-to-treat patient population there was a statistically significant improvement in median OS from 10.4 months on the control arm to 17.4 months on the test arm (Hazard Ratio 0.65, 80% CI 0.46–0.91, *p* = 0.1) [120].

In a phase II, single arm study in 37 patients with metastatic KRAS- or EGFR-mutated, treatment-naïve non-small cell lung cancer (NSCLC), Reolysin was administered IV with paclitaxel and carboplatin [121]. No new safety concerns were observed. Thirty-one of the 35 evaluable patients had clinical benefit; the ORR was 31% (90% 1-sided lower CI) in comparison with the assumed historical response rate for paclitaxel and carboplatin alone of 20%. The median PFS and OS were 4 and 13.1 months respectively and 7 patients (20%) were still alive after a median follow-up of 34.2 months (range 26.9–71.5 months). Thus, it has been suggested that the effects in OS may be the result of the immunogenicity induced by Reolysin against the tumor cells.

REO 017 was a single arm Phase II study of Reolysin with gemcitabine in chemotherapy-naïve patients with advanced pancreatic adenocarcinoma [122]. With a median follow up of 2 years, the median PFS and OS were 4 months and 10.2 months, respectively [122]. Half of patients received chemotherapy after progression, including 12% nab-paclitaxel. On-treatment biopsies revealed virus localization in malignant cells, caspase-3 activation and increased PD-L1 expression in malignant cells [122, 123]. In a randomized Phase II study in a similar patient population, paclitaxel and carboplatin were administered alone (37 patients) or in combination with Reolysin (36 patients) [124, 125]. The median OS time in the test arm was median was 7.3 months (95% CI: 4.8–11.2 months) and in the control arm was 8.8 months (95% CI: 6.6–11.8, (*p* = 0.68). The median PFS time was 4.9 months (95% CI: 3.0–6.3 months) in the test arm versus 5.2 months (95% CI: 2.3–6.2 months) in the control arm (*p* = 0.6). Despite the fact that Noonan et al. did not find any differences in response rate, PFS or OS between the two arms, the mature data showed a possible delayed effect on OS, with a divergence of survival curves occurring around year 1, and the strongest efficacy signal for improvement in OS occurring around year 2 in the Reolysin-containing arms in comparison to control arms. In fact, the estimated 2-year survival probability in the study with Reolysin and gemcitabine is 24% (95% CI 11–39%), consistent with estimated 2-year survival probability of 20% (95% CI 8–36%) in the randomized arm receiving Reolysin and in the NCI-8601 study.

The addition of Reolysin to chemotherapy has given mixed results depending on the tumor studied. What is becoming clear is that Reolysin works more like an immune-therapy agent with a major delayed effect in survival rather than as a cytotoxic that controls response rate and/or progression free survival.

The safety of ITu Reolysin with palliative radiotherapy was evaluated in a phase I study of patients with advanced cancer [126]. Among 23 patients, no DLT was observed, and the most common AEs were fever, flu-like symptoms, vomiting, asymptomatic lymphopenia and neutropenia.

To date, the AEs associated with Reolysin have been mild to moderate in severity and predominantly flu-like in nature (fever, chills, headache, fatigue, rhinorrhea, cough, myalgia, arthralgia, nausea, vomiting and diarrhea). Moderate and transient alterations in hepatic function tests and hematological investigations have also been observed.

#### Coxsackie virus (CXV)

Decay-accelerating factor (DAF) is highly expressed on surfaces of many tumors, including melanoma [127, 128]. A combination of surface-expressed intercellular adhesion molecule-1 (ICAM-1) and DAF forms the cellular receptor complex for CXV A21 (CAV21), resulting in specific viral attachment, cell internalization, and subsequent rapid cell lysis [129]. ITu, IP or IV administration of CVA21 were equally effective in reducing the tumor volume in melanoma xenografts [130]. CAV21 (CAVATAK) has been tested in humans. A phase II study in patients with advanced melanoma (CALM) has been completed [131]. The primary outcome of this study was PFS of more than 16% in 6 months (using the immure-related response criteria). CVA21 was administered by ITu injection in 57 patients. Almost 40% of the patients were alive and progression-free at 6 months, and the study reached its primary endpoint. The ORR using the immune-related response criteria was 28.1% with durable responses in 19.3% of the patients. Treatment was well tolerated with no grade 3/4 agent-related AEs. The most common AEs were grade 1 local injection reactions and flu-like illness.

#### Measles virus (MV)

MV enters cells through interaction of its H protein and cellular CD46 (membrane cofactor protein) and signaling lymphocyte–activating molecule (SLAM) [132–134]. The wild-type MV enter cells more effectively through SLAM; however, the MV vaccine strains enter more effectively via the CD46 receptors [135]. CD46 is overexpressed on tumor cells [136]. MV vaccine affects only cells with a high density of CD46, and therefore, does not affect normal cells [137]. MV kills tumor cells by inducing cell-to-cell fusion through F protein, formation of syncytia and subsequent apoptotic death [137–139]. Several preclinical studies in animal models, including both solid tumors and hematologic malignancies, have evaluated MV through different routes (ITu, IV, IP or intrapleural) and administration schedules [140–144]. Treated animal tumors demonstrate cytopathic effect with syncytia formation followed by apoptotic cell death of MV-infected tumor cells [145].

A phase I study of IP MV-CEA, a carcinoembryonic antigen expressing MV, to patients with recurrent ovarian cancer has been completed [146]. A total of 21 patients with measles immunity and platinum-refractory recurrent ovarian cancer were treated. No DLT was observed; the most common AEs were fever and abdominal pain. The best objective response was SD in 14 patients with a median duration of 92.5 days; clinical outcome was dose-dependent. All patients with viremia or CEA elevation had SD. In another phase I study, MV-NIS – a sodium-iodide symporter (NIS) expressing MV – was administered by IP injection every 4 weeks in patients with platinum-resistant ovarian cancer [147]. Therapy was well tolerated and no DLT was observed in 16 patients treated at high-dose levels. Viral accumulation was confirmed by ^123^I uptake on functional imaging and was associated with long PFS. There was evidence of induction of anti-tumor immunity, as anti-tumor effector T cells recognizing tumor antigens were increased post-treatment. The median OS was 26.5 months. Early reports from a Phase I trial of systemically administered MV-NIS support selective replication within tumor tissue and potential efficacy in multiple myeloma [148]. Both patients in this report were seronegative for measles; decrease in tumor burden was seen in both and was maintained for 9 months in one patient.

#### Newcastle disease virus (NDV)

NDV is not pathogenic to humans and has been extensively studied as an oncolytic agent in several different human tumor cell lines and tumor models [149–150]. NDV binds cells via the hemagglutinin neuraminidase (HN) protein [151]. PV701 and MTH68/H are live attenuated oncolytic viral strains of NDV, which have the capacity to selectively replicate in and lyse tumor cells and to cause immunostimulation [152].

In three Phase I studies, PV701 has been given IV and shown to replicate in tumor cells, resulting in lysis of different tumor types [153]. In a phase I study evaluating four dosing regimens, 79 patients with advanced solid cancers that were unresponsive to standard therapy received PV701 IV [154]. The most common AEs were flu-like symptoms, occurring mostly after the first dose and decreasing in number and severity with each subsequent dose. Tumor site-specific AEs and acute dosing reactions were also observed, without cumulative toxicity. Objective responses occurred at higher dose levels, with PFS of up to 31 months. Electron microscopy of tumor tissue from one patient, 11 months after therapy, demonstrated PV701 particles budding from the tumor cell membrane.

MTH68/H has been given to patients with high-grade glioma although outside of a clinical trial setting. In a report of four cases of advanced high-grade glioma after failure of conventional treatments, treatment with MTH-68/H resulted in survival rates of 5–9 years [155]. NDV-HUJ is a unique strain that can overcome the anti-apoptotic effect of Livin [156]. The phase I/II trial of IV NDV-HUJ in recurrent GBM showed good tolerability and responses [157]. Eleven of 14 patients received treatment with minimal toxicity of grade 1/2 fever in five patients; MTD was not achieved. One patient had a complete response.

#### Seneca valley virus (SVV-001)

Upon infection, this agent replicates intracellularly, resulting in tumor cell lysis and reduced tumor cell proliferation; the selective tropism of virus replication may involve receptor-mediated internalization [158–160]. In small cell lung cancer (SCLC) xenografts, doses ≥10^8^ viral particles/kg resulted in complete and durable eradication of tumors in all mice treated [161]. In a Phase I clinical trial, 30 patients were treated with SVV-001, including six with SCLC [162]. SVV-001 was well tolerated, with no DLT observed in any dose cohort [162]. Viral clearance was documented in all subjects and related temporally with the development of antiviral antibodies. Evidence of *in vivo* intratumoral viral replication was observed among patients with small cell carcinoma. One patient with previously progressive chemorefractory SCLC remained progression-free for 10 months after SVV-001 administration and is alive over 3 years after treatment.

#### Vesicular stomatitis virus (VSV)

rVSVs that express cellular genes, which modulate immunity, such as beta interferon (IFN-β) genes, result in effective oncolytic activity with increased antitumor immune response [163, 164]. VSVs with a mutation of the matrix (M) gene create a protein incapable of blocking mRNA export from the nucleus, resulting in the stimulation of robust innate immune responses following the infection of cancer cells, which increases production IFN-β and interferon-stimulated gene (ISG) [165, 166]. The resultant innate immune response causes stimulates the antigen-presenting machinery, increases NK cell activity, and induces anticancer cytokines. A toxicology study in rats and rhesus macaques has shown that ITu injection of oncolytic VSV expressing human interferon-β (VSV-hIFNβ) did not have any observed AEs [167]. No clinical trial results have been reported as of yet.

### Oncolytic virotherapy: combination therapy and future directions

The generation of an antitumor immune response is an indirect mechanism of malignant cell death for both OV-infected and non-infected cells [34]. There is a theoretical concern that tumor-infiltrating lymphocytes can suppress viral replication and finally eradicate the virus. Pre-existing antibodies can also bind IV administered OV and clear it from the circulation, minimizing viral penetration. Combination strategies of OV with chemotherapeutic agents can potentially overcome those obstacles. Cyclophosphamide has been shown in preclinical animal models to improve reovirus access to the tumor and preserve neutralizing antibody levels sufficient for prevention of severe toxicity [168] but did not appear to affect antibody levels and duration of viremia [169]. Gemcitabine appears to negatively impact late phases of reovirus replication; however, the net effect is synergistic as it accelerates antitumor immunity generation most likely by decreasing immunosuppressive cells within the tumor microenvironment [170]. In a Phase II study of Reolysin in combination with gemcitabine, combination treatment did not prevent viral entry in malignant cells and subsequent apoptosis [122, 123]. Antibody response to Reolysin also appears to be attenuated with this combination strategy [171]. Activation of the programmed death-1/programmed death-ligand 1 (PD-1/PD-L1) axis in tumor cells can be induced by oncolytic virotherapy [122, 172], a finding likely related to natural stimulation of checkpoint molecules in the setting of chronic viral infections in order to minimize tissue damage [173].

There are preclinical data for synergy between OVs and immune checkpoint inhibition. In melanoma xenografts, the combination of Reolysin and anti-PD1 antibody significantly prolonged mice survival compared to either agent alone [174]. There was evidence of enhanced antitumor cytotoxic T cell and natural killer (NK) cell activity with the combination therapy. Suppression of antitumor immunity by regulatory T cells (Treg) in Reolysin alone treated mice was ameliorated by anti-PD1 therapy. In an immunotherapy-resistant lung adenocarcinoma animal model, treatment with oncolytic adenovirus plus anti-PD-1 antibody, significantly increased antitumor immune responses to multiple neoantigens and decreased tumor growth, suggesting reversal of anti-PD-1 resistance with oncolytic virotherapy [175]. NDV combined with immune checkpoint inhibition in immunogenic and non-immunogenic tumor animal models led to increased antitumor immunity and efficacy compared to either agent alone [34]. VSV-mIFNβ-NIS, an oncolytic VSV encoding human murine IFNβ and NIS, it has been shown to be active in an acute myeloid leukemia model and its activity was increased with anti-PD1 antibody therapy [176]. Synergy of oncolytic VSV with anti-PD1 antibody therapy has been also demonstrated in glioma models [177]. An oncolytic measles virus armed with genes coding for antibodies against inhibitory immune checkpoints has been shown to have improved antitumor activity compared to control virus [178]. It appears to be a synergistic effect between oncolytic measles virus and immune checkpoint inhibitors [178]. Synergy of ITu vaccinia virus with ITu immune checkpoint blockade and radiation had been established in a lymphoma xenograft model, with tumor shrinkage in both treated and untreated tumors [179]. The results of oncolytic virotherapy and immune checkpoint inhibition in a Phase I clinical trial are promising [55]. Enhanced viral clearance with checkpoint inhibition still remains a concern [180].

Small molecule inhibitors can potentially improve OV penetration and activity. As mentioned in previous sections, combination strategies of OV with agents targeting the VEGF/VEGFR pathway are based on the potential selective targeting of the tumor neovasculature. VEGFR TKIs can also have off-target effects on antiviral defense mechanisms [28, 29]. Reolysin has shown synergy with VEGFR TKIs *in vitro* and *in vivo* in NSCLC models, with decrease in tumor growth and increase in antitumor immunity [181]. Similar efficacy is observed with BRAF and MEK inhibitors in BRAF mutated melanoma cells through induction of ER stress when combined with BRAF inhibition [182].

Multiple ambiguities exist regarding the optimization of combination strategies. It is unclear when the OV should be administered in regards to other novel agents. For example, administration of checkpoint inhibitors with OV on the same day or subsequent days in clinical trials or preclinical models has been performed but not been compared in a single study. It is not known whether the combination of two different OV has better efficacy compared to single agent therapy. Most importantly, we lack biomarkers predictive of response. Mutations in RAS pathway for Reolysin, Rb pathway mutations for adenovirus, GADD34 mutations for HSV are potential biomarker candidates but to date, no single study has stratified patients based on biomarkers of response. Real-time evidence of enhanced antitumor immune response generation as well as dynamic imaging for tumor perfusion may be methods that predict the benefit from oncolytic virotherapy. For example, in patients treated with T-VEC who develop minimal increase in CD^8+^ T cells from baseline after 6 weeks of therapy, the risk of subsequent disease progression is high [183]. Changes in PD-L1 expression and tumor-infiltrating lymphocytes in tumor tissue over time as well as alterations in serum cytokine profile and gene expression studies are currently being evaluated as secondary outcomes in clinical trials (clinicaltrials.gov NCT02824965, NCT02272855, NCT02364713).

## CONCLUSIONS

After more than 100 years of intense research as a cancer therapeutic, the first OV therapy obtained regulatory approval in 2015, a modest success when compared to multiple cytotoxic agents, small molecule and antibody drug development programs that have obtained approval for clinical use over the last three decades. Although impressively active as anticancer agents *in vitro* and *in vitro*, with a favorable toxicity profile, the effectiveness as single agent therapy with OVs is limited. Thus, the addition of other agents to enhance OV efficacy is necessary. The immune system can enhance viral-induced oncolysis yet decrease viral penetration after systemic administration. Combinations of OV with cytotoxic agents are feasible and safe, with the potential of transient immunosuppression of the host in order to increase viral access to the tumor and provide time for viral oncolysis to exceed the tumor's replicative potential. Despite pre-clinical synergy with anti-angiogenic agents, it still remains to be seen if the combinatory approach would be clinically effective in randomized studies. Excessive antiviral immune response can also potentially eradicate the virus before it reaches its peak effect. Immune checkpoint activation in tumor cells has been noted with oncolytic virotherapy, a finding similar to the observed effector T cell exhaustion during chronic viral infections [184], therefore, providing rationale for combination strategies with novel immunotherapies. The future success of OV is likely to be determined through identification of biomarkers for tumor response that is currently lacking, improvement of OV delivery to tumor and its surrounding microenvironment, and ultimately its ability to enhance anti-tumor immune response through combinatory therapeutic approaches.

## References

[1] Sinkovics JG, Horvath JC (2008). Natural and genetically engineered viral agents for oncolysis and gene therapy of human cancers. Arch Immunol Ther Exp (Warsz).

[2] Hoster HA, Zanes RP, Von Haam E (1949). Studies in Hodgkin's syndrome; the association of viral hepatitis and Hodgkin's disease; a preliminary report. Cancer Res.

[3] Martuza RL, Malick A, Markert JM, Ruffner KL, Coen DM (1991). Experimental therapy of human glioma by means of a genetically engineered virus mutant. Science.

[4] FDA (2015). “FDA approves first-of-its-kind product for the treatment of melanoma.”. http://www.fda.gov/NewsEvents/Newsroom/PressAnnouncements/ucm469571.htm.

[5] EMA (2015). “First oncolytic immunotherapy medicine recommended for approval.”. http://www.ema.europa.eu/ema/index.jsp?curl=pages/news_and_events/news/2015/10/news_detail_002421.jsp&mid=WC0b01ac058004d5c1.

[6] Alain T, Kim TS, Lun X, Liacini A, Schiff LA, Senger DL, Forsyth PA (2007). Proteolytic disassembly is a critical determinant for reovirus oncolysis. Mol Ther.

[7] Marcato P, Shmulevitz M, Pan D, Stoltz D, Lee PW (2007). Ras transformation mediates reovirus oncolysis by enhancing virus uncoating, particle infectivity, and apoptosis-dependent release. Mol Ther.

[8] Strong JE, Coffey MC, Tang D, Sabinin P, Lee PW (1998). The molecular basis of viral oncolysis: usurpation of the Ras signaling pathway by reovirus. EMBO J.

[9] Field HJ, Wildy P (1978). The pathogenicity of thymidine kinase-deficient mutants of herpes simplex virus in mice. J Hyg (Lond).

[10] Jamieson AT, Gentry GA, Subak-Sharpe JH (1974). Induction of both thymidine and deoxycytidine kinase activity by herpes viruses. J Gen Virol.

[11] Whitley RJ, Roizman B (2001). Herpes simplex virus infections. Lancet.

[12] Gasparri F, Wang N, Skog S, Galvani A, Eriksson S (2009). Thymidine kinase 1 expression defines an activated G1 state of the cell cycle as revealed with site-specific antibodies and ArrayScan assays. Eur J Cell Biol.

[13] Verheije MH, Rottier PJ (2012). Retargeting of viruses to generate oncolytic agents. Adv Virol.

[14] Prestwich RJ, Errington F, Ilett EJ, Morgan RS, Scott KJ, Kottke T, Thompson J, Morrison EE, Harrington KJ, Pandha HS, Selby PJ, Vile RG, Melcher AA (2008). Tumor infection by oncolytic reovirus primes adaptive antitumor immunity. Clin Cancer Res.

[15] Toda M, Rabkin SD, Kojima H, Martuza RL (1999). Herpes simplex virus as an in situ cancer vaccine for the induction of specific anti-tumor immunity. Hum Gene Ther.

[16] Toda M, Martuza RL, Kojima H, Rabkin SD (1998). In situ cancer vaccination: an IL-12 defective vector/replication-competent herpes simplex virus combination induces local and systemic antitumor activity. J Immunol.

[17] Thorne SH, Contag CH (2008). Integrating the biological characteristics of oncolytic viruses and immune cells can optimize therapeutic benefits of cell-based delivery. Gene Ther.

[18] Diaconu I, Cerullo V, Hirvinen ML, Escutenaire S, Ugolini M, Pesonen SK, Bramante S, Parviainen S, Kanerva A, Loskog AS, Eliopoulos AG, Pesonen S, Hemminki A (2012). Immune response is an important aspect of the antitumor effect produced by a CD40L-encoding oncolytic adenovirus. Cancer Res.

[19] Miyamoto S, Inoue H, Nakamura T, Yamada M, Sakamoto C, Urata Y, Okazaki T, Marumoto T, Takahashi A, Takayama K, Nakanishi Y, Shimizu H, Tani K (2012). Coxsackievirus B3 is an oncolytic virus with immunostimulatory properties that is active against lung adenocarcinoma. Cancer Res.

[20] Donnelly OG, Errington-Mais F, Steele L, Hadac E, Jennings V, Scott K, Peach H, Phillips RM, Bond J, Pandha H, Harrington K, Vile R, Russell S (2013). Measles virus causes immunogenic cell death in human melanoma. Gene Ther.

[21] Kepp O, Galluzzi L, Martins I, Schlemmer F, Adjemian S, Michaud M, Sukkurwala AQ, Menger L, Zitvogel L, Kroemer G (2011). Molecular determinants of immunogenic cell death elicited by anticancer chemotherapy. Cancer Metastasis Rev.

[22] Breitbach CJ, De Silva NS, Falls TJ, Aladl U, Evgin L, Paterson J, Sun YY, Roy DG, Rintoul JL, Daneshmand M, Parato K, Stanford MM, Lichty BD (2011). Targeting tumor vasculature with an oncolytic virus. Mol Ther.

[23] Benencia F, Courreges MC, Conejo-García JR, Buckanovich RJ, Zhang L, Carroll RH, Morgan MA, Coukos G (2005). Oncolytic HSV exerts direct antiangiogenic activity in ovarian carcinoma. Hum Gene Ther.

[24] Breitbach CJ, Arulanandam R, De Silva N, Thorne SH, Patt R, Daneshmand M, Moon A, Ilkow C, Burke J, Hwang TH, Heo J, Cho M, Chen H (2013). Oncolytic vaccinia virus disrupts tumor-associated vasculature in humans. Cancer Res.

[25] Zhang Z, Zou W, Wang J, Gu J, Dang Y, Li B, Zhao L, Qian C, Qian Q, Liu X (2005). Suppression of tumor growth by oncolytic adenovirus-mediated delivery of an antiangiogenic gene, soluble Flt-1. Mol Ther.

[26] Gholami S, Marano A, Chen NG, Aguilar RJ, Frentzen A, Chen CH, Lou E, Fujisawa S, Eveno C, Belin L, Zanzonico P, Szalay A, Fong Y (2014). A novel vaccinia virus with dual oncolytic and anti-angiogenic therapeutic effects against triple-negative breast cancer. Breast Cancer Res Treat.

[27] Hou W, Chen H, Rojas J, Sampath P, Thorne SH (2014). Oncolytic vaccinia virus demonstrates antiangiogenic effects mediated by targeting of VEGF. Int J Cancer.

[28] Jha BK, Polyakova I, Kessler P, Dong B, Dickerman B, Sen GC, Silverman RH (2011). Inhibition of RNase L and RNA-dependent protein kinase (PKR) by sunitinib impairs antiviral innate immunity. J Biol Chem.

[29] Jha BK, Dong B, Nguyen CT, Polyakova I, Silverman RH (2013). Suppression of antiviral innate immunity by sunitinib enhances oncolytic virotherapy. Mol Ther.

[30] Alemany R, Suzuki K, Curiel DT (2000). Blood clearance rates of adenovirus type 5 in mice. J Gen Virol.

[31] Hu JC, Coffin RS, Davis CJ, Graham NJ, Groves N, Guest PJ, Harrington KJ, James ND, Love CA, McNeish I, Medley LC, Michael A, Nutting CM (2006). A phase I study of OncoVEXGM-CSF, a second-generation oncolytic herpes simplex virus expressing granulocyte macrophage colony-stimulating factor. Clin Cancer Res.

[32] Andtbacka RH, Kaufman HL, Collichio F, Amatruda T, Senzer N, Chesney J, Delman KA, Spitler LE, Puzanov I, Agarwala SS, Milhem M, Cranmer L, Curti B (2015). Talimogene Laherparepvec Improves Durable Response Rate in Patients With Advanced Melanoma. J Clin Oncol.

[33] Senzer NN, Kaufman HL, Amatruda T, Nemunaitis M, Reid T, Daniels G, Gonzalez R, Glaspy J, Whitman E, Harrington K, Goldsweig H, Marshall T, Love C (2009). Phase II clinical trial of a granulocyte-macrophage colony-stimulating factor-encoding, second-generation oncolytic herpesvirus in patients with unresectable metastatic melanoma. J Clin Oncol.

[34] Zamarin D, Holmgaard RB, Subudhi SK, Park JS, Mansour M, Palese P, Merghoub T, Wolchok JD, Allison JP (2014). Localized Oncolytic Virotherapy Overcomes Systemic Tumor Resistance to Immune Checkpoint Blockade Immunotherapy. Sci Transl Med.

[35] Dubin G, Fishman NO, Eisenberg RJ, Cohen GH, Friedman HM (1992). The role of herpes simplex virus glycoproteins in immune evasion. Curr Top Microbiol Immunol.

[36] Whitley R, Fields BN, Howley PM, Chanock RM, Melnick JL, Monath TP, Roizman B, Straus SE (1996). Herpes simplex viruses. Fields Virology.

[37] Tyler KL, Fields BN, Fields BN, Knipe DM, Chanock RM, Hirsch MS, Melnick JL, Monath TP, Roizman B (1990). Reoviruses.

[38] Wakimoto H, Johnson PR, Knipe DM, Chiocca EA (2003). Effects of innate immunity on herpes simplex virus and its ability to kill tumor cells. Gene Ther.

[39] Toda M, Martuza RL, Rabkin SD (2000). Tumor growth inhibition by intratumoral inoculation of defective herpes simplex virus vectors expressing granulocyte-macrophage colony-stimulating factor. Mol Ther.

[40] Bolovan CA, Sawtell NM, Thompson RL (1994). ICP34.5 mutants of herpes simplex virus type 1 strain 17syn+ are attenuated for neurovirulence in mice and for replication in confluent primary mouse embryo cell cultures. J Virol.

[41] Brown SM, MacLean AR, Aitken JD, Harland J (1994). ICP34.5 influences herpes simplex virus type 1 maturation and egress from infected cells in vitro. J Gen Virol.

[42] He B, Gross M, Roizman B (1997). The gamma(1)34.5 protein of herpes simplex virus 1 complexes with protein phosphatase 1alpha to dephosphorylate the alpha subunit of the eukaryotic translation initiation factor 2 and preclude the shutoff of protein synthesis by double-stranded RNA-activated protein kinase. Proc Natl Acad Sci USA.

[43] Chou J, Kern ER, Whitley RJ, Roizman B (1990). Mapping of herpes simplex virus-1 neurovirulence to gamma 134.5, a gene nonessential for growth in culture. Science.

[44] Chou J, Chen JJ, Gross M, Roizman B (1995). Association of a M(r) 90,000 phosphoprotein with protein kinase PKR in cells exhibiting enhanced phosphorylation of translation initiation factor eIF-2 alpha and premature shutoff of protein synthesis after infection with gamma 134.5- mutants of herpes simplex virus 1. Proc Natl Acad Sci USA.

[45] He B, Chou J, Liebermann DA, Hoffman B, Roizman B (1996). The carboxyl terminus of the murine MyD116 gene substitutes for the corresponding domain of the gamma(1)34.5 gene of herpes simplex virus to preclude the premature shutoff of total protein synthesis in infected human cells. J Virol.

[46] Su ZZ, Emdad L, Sarkar D, Randolph A, Valerie K, Yacoub A, Dent P, Fisher PB (2005). Potential molecular mechanism for rodent tumorigenesis: mutational generation of Progression Elevated Gene-3 (PEG-3). Oncogene.

[47] McKie EA, MacLean AR, Lewis AD, Cruickshank G, Rampling R, Barnett SC, Kennedy PG, Brown SM (1996). Selective in vitro replication of herpes simplex virus type 1 (HSV-1) ICP34.5 null mutants in primary human CNS tumours—evaluation of a potentially effective clinical therapy. Br J Cancer.

[48] MacLean AR, ul-Fareed M, Robertson L, Harland J, Brown SM (1991). Herpes simplex virus type 1 deletion variants 1714 and 1716 pinpoint neurovirulence-related sequences in Glasgow strain 17+ between immediate early gene 1 and the ‘a’ sequence. J Gen Virol.

[49] Mulvey M, Poppers J, Ladd A, Mohr I (1999). A herpesvirus ribosome-associated, RNA-binding protein confers a growth advantage upon mutants deficient in a GADD34-related function. J Virol.

[50] Früh K, Ahn K, Djaballah H, Sempé P, van Endert PM, Tampé R, Peterson PA, Yang Y (1995). A viral inhibitor of peptide transporters for antigen presentation. Nature.

[51] York IA, Roop C, Andrews DW, Riddell SR, Graham FL, Johnson DC (1994). A cytosolic herpes simplex virus protein inhibits antigen presentation to CD8+ T lymphocytes. Cell.

[52] Wolchok JD, Hoos A, O’Day S, Weber JS, Hamid O, Lebbé C, Maio M, Binder M, Bohnsack O, Nichol G, Humphrey R, Hodi FS (2009). Guidelines for the evaluation of immune therapy activity in solid tumors: immune-related response criteria. Clin Cancer Res.

[53] Eisenhauer EA, Therasse P, Bogaerts J, Schwartz LH, Sargent D, Ford R, Dancey J, Arbuck S, Gwyther S, Mooney M, Rubinstein L, Shankar L, Dodd L (2009). New response evaluation criteria in solid tumours: revised RECIST guideline (version 1.1). Eur J Cancer.

[54] Puzanov I, Milhem MM, Andtbacka RH, Minor DR, Hamid O, Li A, Chastain M, Gorski K, Anderson A, Vanderwalde AM, Chou J, Kaufman H (2014). Primary analysis of a phase 1b multicenter trial to evaluate safety and efficacy of talimogene laherparepvec (T-VEC) and ipilimumab (ipi) in previously untreated, unresected stage IIIB-IV melanoma. ASCO Meeting Abstracts.

[55] Puzanov I, Milhem MM, Andtbacka RH, Minor DR, Hamid O, Li A, Chou J, Kaufman H (2015). Survival, safety, and response patterns in a phase 1b multicenter trial of talimogene laherparepvec (T-VEC) and ipilimumab (ipi) in previously untreated, unresected stage IIIB-IV melanoma. ASCO Meeting Abstracts.

[56] Long GV, Dummer R, Ribas A, Puzanov I, Michielin O, Vanderwalde A, Andtbacka RH, Cebon J, Fernandez E, Malvehy J, Olszanski AJ, Gajewski TF, Kirkwood JM (2015). 24LBA Safety data from the phase 1b part of the MASTERKEY-265 study combining talimogene laherparepvec (T-VEC) and pembrolizumab for unresectable stage IIIB-IV melanoma. Eur J Cancer.

[57] Ribas A, Puzanov I, Gajewski T, Long GV, Dummer R, Kirkwood JM, VanderWalde A, Cebon JS, McArthur GA, Gause CK, Chen L, Kaufman DR, Chou J (2015). A multicenter, open-label trial of talimogene laherparepvec (T-VEC) plus pembrolizumab vs pembrolizumab monotherapy in previously untreated, unresected, stage IIIB-IV melanoma. ASCO Meeting Abstracts.

[58] Long GV, Dummer R, Ribas A, Puzanov I, VanderWalde A, Andtbacka RH, Michielin O, Olszanski AJ, Malvehy J, Cebon JS, Fernandez E, Kirkwood JM, Gajewski T (2016). Efficacy analysis of MASTERKEY-265 phase 1b study of talimogene laherparepvec (T-VEC) and pembrolizumab (pembro) for unresectable stage IIIB-IV melanoma. ASCO Meeting Abstracts.

[59] Homa FL, Brown JC (1997). Capsid assembly and DNA packaging in herpes simplex virus. Rev Med Virol.

[60] Filippakis H, Spandidos DA, Sourvinos G (2010). Herpesviruses: hijacking the Ras signaling pathway. Biochim Biophys Acta.

[61] Roizman B, Markovitz N (1997). Herpes simplex virus virulence: the functions of the gamma (1)34.5 gene. J Neurovirol.

[62] Mineta T, Rabkin SD, Yazaki T, Hunter WD, Martuza RL (1995). Attenuated multi-mutated herpes simplex virus-1 for the treatment of malignant gliomas. Nat Med.

[63] Goldstein DJ, Weller SK (1988). Factor(s) present in herpes simplex virus type 1-infected cells can compensate for the loss of the large subunit of the viral ribonucleotide reductase: characterization of an ICP6 deletion mutant. Virology.

[64] Mineta T, Rabkin SD, Martuza RL (1994). Treatment of malignant gliomas using ganciclovir-hypersensitive, ribonucleotide reductase-deficient herpes simplex viral mutant. Cancer Res.

[65] Varghese S, Rabkin SD (2002). Oncolytic herpes simplex virus vectors for cancer virotherapy. Cancer Gene Ther.

[66] Langelier Y, Champoux L, Hamel M, Guilbault C, Lamarche N, Gaudreau P, Massie B (1998). The R1 subunit of herpes simplex virus ribonucleotide reductase is a good substrate for host cell protein kinases but is not itself a protein kinase. J Biol Chem.

[67] Markert JM, Medlock MD, Rabkin SD, Gillespie GY, Todo T, Hunter WD, Palmer CA, Feigenbaum F, Tornatore C, Tufaro F, Martuza RL (2000). Conditionally replicating herpes simplex virus mutant, G207 for the treatment of malignant glioma: results of a phase I trial. Gene Ther.

[68] Markert JM, Liechty PG, Wang W, Gaston S, Braz E, Karrasch M, Nabors LB, Markiewicz M, Lakeman AD, Palmer CA, Parker JN, Whitley RJ, Gillespie GY (2009). Phase Ib trial of mutant herpes simplex virus G207 inoculated pre-and post-tumor resection for recurrent GBM. Mol Ther.

[69] Markert JM, Razdan SN, Kuo HC, Cantor A, Knoll A, Karrasch M, Nabors LB, Markiewicz M, Agee BS, Coleman JM, Lakeman AD, Palmer CA, Parker JN (2014). A phase 1 trial of oncolytic HSV-1, G207, given in combination with radiation for recurrent GBM demonstrates safety and radiographic responses. Mol Ther.

[70] Berkowitz C, Moyal M, Rösen-Wolff A, Darai G, Becker Y (1994). Herpes simplex virus type 1 (HSV-1) UL56 gene is involved in viral intraperitoneal pathogenicity to immunocompetent mice. Arch Virol.

[71] Roizman B (1996). The function of herpes simplex virus genes: a primer for genetic engineering of novel vectors. Proc Natl Acad Sci USA.

[72] Kemeny N, Brown K, Covey A, Kim T, Bhargava A, Brody L, Guilfoyle B, Haag NP, Karrasch M, Glasschroeder B, Knoll A, Getrajdman G, Kowal KJ (2006). Phase I, open-label, dose-escalating study of a genetically engineered herpes simplex virus, NV1020, in subjects with metastatic colorectal carcinoma to the liver. Hum Gene Ther.

[73] Fong Y, Kim T, Bhargava A, Schwartz L, Brown K, Brody L, Covey A, Karrasch M, Getrajdman G, Mescheder A, Jarnagin W, Kemeny N (2009). A herpes oncolytic virus can be delivered via the vasculature to produce biologic changes in human colorectal cancer. Mol Ther.

[74] Fujimoto Y, Mizuno T, Sugiura S, Goshima F, Kohno S, Nakashima T, Nishiyama Y (2006). Intratumoral injection of herpes simplex virus HF10 in recurrent head and neck squamous cell carcinoma. Acta Otolaryngol.

[75] Kimata H, Imai T, Kikumori T, Teshigahara O, Nagasaka T, Goshima F, Nishiyama Y, Nakao A (2006). Pilot study of oncolytic viral therapy using mutant herpes simplex virus (HF10) against recurrent metastatic breast cancer. Ann Surg Oncol.

[76] Nakao A, Kasuya H, Sahin TT, Nomura N, Kanzaki A, Misawa M, Shirota T, Yamada S, Fujii T, Sugimoto H, Shikano T, Nomoto S, Takeda S (2011). A phase I dose-escalation clinical trial of intraoperative direct intratumoral injection of HF10 oncolytic virus in non-resectable patients with advanced pancreatic cancer. Cancer Gene Ther.

[77] Ferris RL, Gross ND, Nemunaitis JJ, Andtbacka RH, Argiris A, Ohr J, Vetto JT, Senzer NN, Bedell C, Ungerleider RS, Tanaka M, Nishiyama Y (2014). Phase I trial of intratumoral therapy using HF10, an oncolytic HSV-1, demonstrates safety in HSV+/HSV- patients with refractory and superficial cancers. ASCO Meeting Abstracts.

[78] Mastrangelo MJ, Maguire HC, Eisenlohr LC, Laughlin CE, Monken CE, McCue PA, Kovatich AJ, Lattime EC (1999). Intratumoral recombinant GM-CSF-encoding virus as gene therapy in patients with cutaneous melanoma. Cancer Gene Ther.

[79] Park BH, Hwang T, Liu TC, Sze DY, Kim JS, Kwon HC, Oh SY, Han SY, Yoon JH, Hong SH, Moon A, Speth K, Park C (2008). Use of a targeted oncolytic poxvirus, JX-594, in patients with refractory primary or metastatic liver cancer: a phase I trial. Lancet Oncol.

[80] Hwang TH, Moon A, Burke J, Ribas A, Stephenson J, Breitbach CJ, Daneshmand M, De Silva N, Parato K, Diallo JS, Lee YS, Liu TC, Bell JC, Kirn DH (2011). A mechanistic proof-of-concept clinical trial with JX-594, a targeted multi-mechanistic oncolytic poxvirus, in patients with metastatic melanoma. Mol Ther.

[81] Heo J, Breitbach CJ, Moon A, Kim CW, Patt R, Kim MK, Lee YK, Oh SY, Woo HY, Parato K, Rintoul J, Falls T, Hickman T (2011). Sequential therapy with JX-594, a targeted oncolytic poxvirus, followed by sorafenib in hepatocellular carcinoma: preclinical and clinical demonstration of combination efficacy. Mol Ther.

[82] Gavanier M, Ayav A, Sellal C, Orry X, Claudon M, Bronowicki JP, Laurent V (2016). CT imaging findings in patients with advanced hepatocellular carcinoma treated with sorafenib: Alternative response criteria (Choi, European Association for the Study of the Liver, and modified Response Evaluation Criteria in Solid Tumor (mRECIST)) versus RECIST 1.1. Eur J Radiol.

[83] Choi H, Charnsangavej C, Faria SC, Macapinlac HA, Burgess MA, Patel SR, Chen LL, Podoloff DA, Benjamin RS (2007). Correlation of computed tomography and positron emission tomography in patients with metastatic gastrointestinal stromal tumor treated at a single institution with imatinib mesylate: proposal of new computed tomography response criteria. J Clin Oncol.

[84] Heo J, Reid T, Ruo L, Breitbach CJ, Rose S, Bloomston M, Cho M, Lim HY, Chung HC, Kim CW, Burke J, Lencioni R, Hickman T (2013). Randomized dose-finding clinical trial of oncolytic immunotherapeutic vaccinia JX-594 in liver cancer. Nat Med.

[85] Breitbach CJ, Burke J, Jonker D, Stephenson J, Haas AR, Chow LQ, Nieva J, Hwang TH, Moon A, Patt R, Pelusio A, Le Boeuf F, Burns J (2011). Intravenous delivery of a multi-mechanistic cancer-targeted oncolytic poxvirus in humans. Nature.

[86] Lee J, Park YS, Burke J, Lim HY, Lee J, Kang WK, Park JO, Pelusio A, Breitbach C, Kirn DH (2013). Phase Ib dose-escalation study of Pexa-Vec (pexastimogene devacirepvec; JX-594), an oncolytic and immunotherapeutic vaccinia virus, administered by intravenous (IV) infusions in patients with metastatic colorectal carcinoma (mCRC). ASCO Meeting Abstracts.

[87] Heo J, Chao Y, Jonker DJ, Baron AD, Habersetzer F, Burke J, Breitbach C, Patt RH, Lencioni R, Homerin M, Limacher JM, Lusky M, Hickman T (2013). Phase IIb randomized trial of Pexa-Vec (pexastimogene devacirepvec; JX-594), a targeted oncolytic vaccinia virus, plus best supportive care (BSC) versus BSC alone in patients with advanced hepatocellular carcinoma who have failed sorafenib treatment (TRAVERSE). ASCO Meeting Abstracts.

[88] Habib N, Salama H, Abd El Latif Abu Median A, Isac Anis I, Abd Al Aziz RA, Sarraf C, Mitry R, Havlik R, Seth P, Hartwigsen J, Bhushan R, Nicholls J, Jensen S (2002). Clinical trial of E1B-deleted adenovirus (dl1520) gene therapy for hepatocellular carcinoma. Cancer Gene Ther.

[89] Lu W, Zheng S, Li XF, Huang JJ, Zheng X, Li Z (2004). Intra-tumor injection of H101, a recombinant adenovirus, in combination with chemotherapy in patients with advanced cancers: a pilot phase II clinical trial. World J Gastroenterol.

[90] Heise C, Ganly I, Kim YT, Sampson-Johannes A, Brown R, Kirn D (2000). Efficacy of a replication-selective adenovirus against ovarian carcinomatosis is dependent on tumor burden, viral replication and p53 status. Gene Ther.

[91] Rothmann T, Hengstermann A, Whitaker NJ, Scheffner M, zur Hausen H (1998). Replication of ONYX-015, a potential anticancer adenovirus, is independent of p53 status in tumor cells. J Virol.

[92] Khuri FR, Nemunaitis J, Ganly I, Arseneau J, Tannock IF, Romel L, Gore M, Ironside J, MacDougall RH, Heise C, Randlev B, Gillenwater AM, Bruso P (2000). a controlled trial of intratumoral ONYX-015, a selectively-replicating adenovirus, in combination with cisplatin and 5-fluorouracil in patients with recurrent head and neck cancer. Nat Med.

[93] Garber K (2006). China approves world's first oncolytic virus therapy for cancer treatment. J Natl Cancer Inst.

[94] Ramesh N, Ge Y, Ennist DL, Zhu M, Mina M, Ganesh S, Reddy PS, Yu DC (2006). CG0070, a conditionally replicating granulocyte macrophage colony-stimulating factor—armed oncolytic adenovirus for the treatment of bladder cancer. Clin Cancer Res.

[95] Zwicker J, Müller R (1995). Cell cycle-regulated transcription in mammalian cells. Prog Cell Cycle Res.

[96] Burke JM, Lamm DL, Meng MV, Nemunaitis JJ, Stephenson JJ, Arseneau JC, Aimi J, Lerner S, Yeung AW, Kazarian T, Maslyar DJ, McKiernan JM (2012). A first in human phase 1 study of CG0070, a GM-CSF expressing oncolytic adenovirus, for the treatment of nonmuscle invasive bladder cancer. J Urol.

[97] Bauder B, Suchy A, Gabler C, Weissenböck H (2000). Apoptosis in feline panleukopenia and canine parvovirus enteritis. J Vet Med B Infect Dis Vet Public Health.

[98] Ozawa K, Ayub J, Kajigaya S, Shimada T, Young N (1988). The gene encoding the nonstructural protein of B19 (human) parvovirus may be lethal in transfected cells. J Virol.

[99] Rayet B, Lopez-Guerrero JA, Rommelaere J, Dinsart C (1998). Induction of programmed cell death by parvovirus H-1 in U937 cells: connection with the tumor necrosis factor alpha signalling pathway. J Virol.

[100] Morey AL, Ferguson DJ, Fleming KA (1993). Ultrastructural features of fetal erythroid precursors infected with parvovirus B19 in vitro: evidence of cell death by apoptosis. J Pathol.

[101] Telerman A, Tuynder M, Dupressoir T, Robaye B, Sigaux F, Shaulian E, Oren M, Rommelaere J, Amson R (1993). A model for tumor suppression using H-1 parvovirus. Proc Natl Acad Sci USA.

[102] Geletneky K, Huesing J, Dahm M, Krebs O, Huber B, Capper D, Rommelaere J, Hajda J, Unterberg A (2014). First combined intravenous and intracerebral application of an oncolytic virus, parvoviras h-1, in a phase I/IIa clinical trial in patients with recurrent glioblastoma multiforme (ParvOryx01). J Clin Oncol.

[103] Geletneky K, Angelova A, Leuchs B, Bhat R, Just A, Capper D, Krebs O, Dahm M, Huber B, Unterberg A, Hajda J, Rommelaere J (2014). Combination of intravenous and intracerebral injection of oncolytic parvovirus H-1 in a phase I/IIA clinical trial of patients with recurrent glioblastoma multiforme: penetration of H-1 virus across the blood-brain barrier. Neuro-oncol.

[104] Gong J, Mita MM (2014). Activated ras signaling pathways and reovirus oncolysis: an update on the mechanism of preferential reovirus replication in cancer cells. Front Oncol.

[105] Norman KL, Hirasawa K, Yang AD, Shields MA, Lee PW (2004). Reovirus oncolysis: the Ras/RalGEF/p38 pathway dictates host cell permissiveness to reovirus infection. Proc Natl Acad Sci USA.

[106] Pan D, Pan LZ, Hill R, Marcato P, Shmulevitz M, Vassilev LT, Lee PW (2011). Stabilisation of p53 enhances reovirus-induced apoptosis and virus spread through p53-dependent NF-κB activation. Br J Cancer.

[107] Sei S, Yang Q, Mussio J, Coffey M, Parchment R, Shoemaker R, Tomaszewski J (2006). Synergistic antitumor activity of oncolytic reovirus and chemotherapeutic agents against non-small cell lung cancer. Eur J Cancer, Suppl.

[108] Adair RA, Scott KJ, Fraser S, Errington-Mais F, Pandha H, Coffey M, Selby P, Cook GP, Vile R, Harrington KJ, Toogood G, Melcher AA (2013). Cytotoxic and immune-mediated killing of human colorectal cancer by reovirus-loaded blood and liver mononuclear cells. Int J Cancer.

[109] Gujar SA, Lee PW (2014). Oncolytic virus-mediated reversal of impaired tumor antigen presentation. Front Oncol.

[110] Hall K, Scott KJ, Rose A, Desborough M, Harrington K, Pandha H, Parrish C, Vile R, Coffey M, Bowen D, Errington-Mais F, Melcher AA (2012). Reovirus-mediated cytotoxicity and enhancement of innate immune responses against acute myeloid leukemia. Biores Open Access.

[111] Wadler S, Yu B, Lane M, Klampfer L, Sasazuki T, Shirasawa S, Coffey M (2004). The oncolytic reovirus, REOLYSIN®, augments the anticancer effects of cytotoxic agents in vitro against the ras-mutated human colon cancer cell line HCT116. Eur J Cancer.

[112] Hamano S, Mori Y, Aoyama M, Kataoka H, Tanaka M, Ebi M, Kubota E, Mizoshita T, Tanida S, Johnston RN, Asai K, Joh T (2015). Oncolytic reovirus combined with trastuzumab enhances antitumor efficacy through TRAIL signaling in human HER2-positive gastric cancer cells. Cancer Lett.

[113] Pandha HS, Heinemann L, Simpson GR, Melcher A, Prestwich R, Errington F, Coffey M, Harrington KJ, Morgan R (2009). Synergistic effects of oncolytic reovirus and cisplatin chemotherapy in murine malignant melanoma. Clin Cancer Res.

[114] Sei S, Mussio JK, Yang QE, Nagashima K, Parchment RE, Coffey MC, Shoemaker RH, Tomaszewski JE (2009). Synergistic antitumor activity of oncolytic reovirus and chemotherapeutic agents in non-small cell lung cancer cells. Mol Cancer.

[115] Hirasawa K, Nishikawa SG, Norman KL, Coffey MC, Thompson BG, Yoon CS, Waisman DM, Lee PW (2003). Systemic reovirus therapy of metastatic cancer in immune-competent mice. Cancer Res.

[116] Etoh T, Himeno Y, Matsumoto T, Aramaki M, Kawano K, Nishizono A, Kitano S (2003). Oncolytic viral therapy for human pancreatic cancer cells by reovirus. Clin Cancer Res.

[117] Carew JS, Espitia CM, Zhao W, Kelly KR, Coffey M, Freeman JW, Nawrocki ST (2013). Reolysin is a novel reovirus-based agent that induces endoplasmic reticular stress-mediated apoptosis in pancreatic cancer. Cell Death Dis.

[118] Hirano S, Etoh T, Okunaga R, Shibata K, Ohta M, Nishizono A, Kitano S (2009). Reovirus inhibits the peritoneal dissemination of pancreatic cancer cells in an immunocompetent animal model. Oncol Rep.

[119] Himeno Y, Etoh T, Matsumoto T, Ohta M, Nishizono A, Kitano S (2005). Efficacy of oncolytic reovirus against liver metastasis from pancreatic cancer in immunocompetent models. Int J Oncol.

[120] Bernstein V, Ellard S, Dent SF, Gelmon KA, Dhesy-Thind SK, Mates M, Salim M, Panasci L, Song X, Clemons M, Tu D, Hagerman LJ, Seymour L (2017). CT131 / 12 - A randomized (RCT) phase II study of oncolytic reovirus (pelareorep) plus standard weekly paclitaxel (P) as therapy for metastatic breast cancer (mBC). AACR 108th Annual Meeting.

[121] Villalona-Calero MA, Lam E, Otterson GA, Zhao W, Timmons M, Subramaniam D, Hade EM, Gill GM, Coffey M, Selvaggi G, Bertino E, Chao B, Knopp MV (2016). Oncolytic reovirus in combination with chemotherapy in metastatic or recurrent non-small cell lung cancer patients with KRAS-activated tumors. Cancer.

[122] Mahalingam D, Goel S, Coffey M, Noronha N, Selvaggi G, Nawrocki S, Nuovo G, Mita M (2015). Oncolytic Virus Therapy in Pancreatic Cancer: Clinical Efficacy and Pharmacodynamic Analysis of REOLYSIN in Combination with Gemcitabine in Patients with Advanced Pancreatic Adenocarcinoma. Ann Oncol.

[123] Mahalingam D, Patel S, Nuovo G, Gill G, Selvaggi G, Coffey M, Nawrocki ST (2015). The combination of intravenous Reolysin and gemcitabine induces reovirus replication and endoplasmic reticular stress in a patient with KRAS-activated pancreatic cancer. BMC Cancer.

[124] Bekaii-Saab T, Noonan AM, Lesinski G, Mikhail S, Ciombor K, Pant S, Aparo S, Tahiri S, Thompson A, Sexton J, Marshall JL, Mace T, Wu C (2014). LBA19A multi-institutional randomized phase 2 trial of the oncolytic virus reolysin in the first line treatment metastatic adenocarcinoma of the pancreas (MAP). Ann Oncol.

[125] Noonan AM, Farren MR, Geyer SM, Huang Y, Tahiri S, Ahn D, Mikhail S, Ciombor KK, Pant S, Aparo S, Sexton J, Marshall JL, Mace TA (2016). Randomized Phase 2 Trial of the Oncolytic Virus Pelareorep (Reolysin) in Upfront Treatment of Metastatic Pancreatic Adenocarcinoma. Mol Ther.

[126] Harrington KJ, Karapanagiotou EM, Roulstone V, Twigger KR, White CL, Vidal L, Beirne D, Prestwich R, Newbold K, Ahmed M, Thway K, Nutting CM, Coffey M (2010). Two-stage phase I dose-escalation study of intratumoral reovirus type 3 dearing and palliative radiotherapy in patients with advanced cancers. Clin Cancer Res.

[127] Cheung NK, Walter EI, Smith-Mensah WH, Ratnoff WD, Tykocinski ML, Medof ME (1988). Decay-accelerating factor protects human tumor cells from complement-mediated cytotoxicity in vitro. J Clin Invest.

[128] Li L, Spendlove I, Morgan J, Durrant LG (2001). CD55 is over-expressed in the tumour environment. Br J Cancer.

[129] Shafren DR, Dorahy DJ, Ingham RA, Burns GF, Barry RD (1997). Coxsackievirus A21 binds to decay-accelerating factor but requires intercellular adhesion molecule 1 for cell entry. J Virol.

[130] Au GG, Lindberg AM, Barry RD, Shafren DR (2005). Oncolysis of vascular malignant human melanoma tumors by Coxsackievirus A21. Int J Oncol.

[131] Andtbacka RH, Curti BD, Kaufman H, Daniels GA, Nemunaitis JJ, Spitler LE, Hallmeyer S, Lutzky J, Schultz SM, Whitman ED, Zhou K, Karpathy R, Weisberg JI (2015). Final data from CALM: A phase II study of Coxsackievirus A21 (CVA21) oncolytic virus immunotherapy in patients with advanced melanoma. ASCO Meeting Abstracts.

[132] Tatsuo H, Ono N, Tanaka K, Yanagi Y (2000). SLAM (CDw150) is a cellular receptor for measles virus. Nature.

[133] Naniche D, Varior-Krishnan G, Cervoni F, Wild TF, Rossi B, Rabourdin-Combe C, Gerlier D (1993). Human membrane cofactor protein (CD46) acts as a cellular receptor for measles virus. J Virol.

[134] Dörig RE, Marcil A, Chopra A, Richardson CD (1993). The human CD46 molecule is a receptor for measles virus (Edmonston strain). Cell.

[135] Yanagi Y (2001). [The cellular receptor for measles virus]. [Article in Japanese]. Uirusu.

[136] Fishelson Z, Donin N, Zell S, Schultz S, Kirschfink M (2003). Obstacles to cancer immunotherapy: expression of membrane complement regulatory proteins (mCRPs) in tumors. Mol Immunol.

[137] Anderson BD, Nakamura T, Russell SJ, Peng KW (2004). High CD46 receptor density determines preferential killing of tumor cells by oncolytic measles virus. Cancer Res.

[138] Wild TF, Malvoisin E, Buckland R (1991). Measles virus: both the haemagglutinin and fusion glycoproteins are required for fusion. J Gen Virol.

[139] Galanis E, Bateman A, Johnson K, Diaz RM, James CD, Vile R, Russell SJ (2001). Use of viral fusogenic membrane glycoproteins as novel therapeutic transgenes in gliomas. Hum Gene Ther.

[140] Grote D, Russell SJ, Cornu TI, Cattaneo R, Vile R, Poland GA, Fielding AK (2001). Live attenuated measles virus induces regression of human lymphoma xenografts in immunodeficient mice. Blood.

[141] Iankov ID, Msaouel P, Allen C, Federspiel MJ, Bulur PA, Dietz AB, Gastineau D, Ikeda Y, Ingle JN, Russell SJ, Galanis E (2010). Demonstration of anti-tumor activity of oncolytic measles virus strains in a malignant pleural effusion breast cancer model. Breast Cancer Res Treat.

[142] Blechacz B, Splinter PL, Greiner S, Myers R, Peng KW, Federspiel MJ, Russell SJ, LaRusso NF (2006). Engineered measles virus as a novel oncolytic viral therapy system for hepatocellular carcinoma. Hepatology.

[143] Peng KW, Ahmann GJ, Pham L, Greipp PR, Cattaneo R, Russell SJ (2001). Systemic therapy of myeloma xenografts by an attenuated measles virus. Blood.

[144] Peng KW, TenEyck CJ, Galanis E, Kalli KR, Hartmann LC, Russell SJ (2002). Intraperitoneal therapy of ovarian cancer using an engineered measles virus. Cancer Res.

[145] Zhang SC, Wang WL, Cai WS, Jiang KL, Yuan ZW (2012). Engineered measles virus Edmonston strain used as a novel oncolytic viral system against human hepatoblastoma. BMC Cancer.

[146] Galanis E, Hartmann LC, Cliby WA, Long HJ, Peethambaram PP, Barrette BA, Kaur JS, Haluska PJ, Aderca I, Zollman PJ, Sloan JA, Keeney G, Atherton PJ (2010). Phase I trial of intraperitoneal administration of an oncolytic measles virus strain engineered to express carcinoembryonic antigen for recurrent ovarian cancer. Cancer Res.

[147] Galanis E, Atherton PJ, Maurer MJ, Knutson KL, Dowdy SC, Cliby WA, Haluska P, Long HJ, Oberg A, Aderca I, Block MS, Bakkum-Gamez J, Federspiel MJ (2015). Oncolytic measles virus expressing the sodium iodide symporter to treat drug-resistant ovarian cancer. Cancer Res.

[148] Russell SJ, Federspiel MJ, Peng KW, Tong C, Dingli D, Morice WG, Lowe V, O’Connor MK, Kyle RA, Leung N, Buadi FK, Rajkumar SV, Gertz MA (2014). Remission of disseminated cancer after systemic oncolytic virotherapy. Mayo Clin Proc.

[149] Reichard KW, Lorence RM, Cascino CJ, Peeples ME, Walter RJ, Fernando MB, Reyes HM, Greager JA (1992). Newcastle disease virus selectively kills human tumor cells. J Surg Res.

[150] Lorence RM, Reichard KW, Katubig BB, Reyes HM, Phuangsab A, Mitchell BR, Cascino CJ, Walter RJ, Peeples ME (1994). Complete regression of human neuroblastoma xenografts in athymic mice after local Newcastle disease virus therapy. J Natl Cancer Inst.

[151] Park MS, García-Sastre A, Cros JF, Basler CF, Palese P (2003). Newcastle disease virus V protein is a determinant of host range restriction. J Virol.

[152] Fournier P, Bian H, Szeberényi J, Schirrmacher V (2012). Analysis of three properties of Newcastle disease virus for fighting cancer: tumor-selective replication, antitumor cytotoxicity, and immunostimulation. Methods Mol Biol.

[153] Lorence RM, Pecora AL, Major PP, Hotte SJ, Laurie SA, Roberts MS, Groene WS, Bamat MK (2003). Overview of phase I studies of intravenous administration of PV701, an oncolytic virus. Curr Opin Mol Ther.

[154] Pecora AL, Rizvi N, Cohen GI, Meropol NJ, Sterman D, Marshall JL, Goldberg S, Gross P, O’Neil JD, Groene WS, Roberts MS, Rabin H, Bamat MK, Lorence RM (2002). Phase I trial of intravenous administration of PV701, an oncolytic virus, in patients with advanced solid cancers. J Clin Oncol.

[155] Csatary LK, Gosztonyi G, Szeberenyi J, Fabian Z, Liszka V, Bodey B, Csatary CM (2004). MTH-68/H oncolytic viral treatment in human high-grade gliomas. J Neurooncol.

[156] Lazar I, Yaacov B, Shiloach T, Eliahoo E, Kadouri L, Lotem M, Perlman R, Zakay-Rones Z, Panet A, Ben-Yehuda D (2010). The oncolytic activity of Newcastle disease virus NDV-HUJ on chemoresistant primary melanoma cells is dependent on the proapoptotic activity of the inhibitor of apoptosis protein Livin. J Virol.

[157] Freeman AI, Zakay-Rones Z, Gomori JM, Linetsky E, Rasooly L, Greenbaum E, Rozenman-Yair S, Panet A, Libson E, Irving CS, Galun E, Siegal T (2006). Phase I/II trial of intravenous NDV-HUJ oncolytic virus in recurrent glioblastoma multiforme. Mol Ther.

[158] Venkataraman S, Reddy SP, Loo J, Idamakanti N, Hallenbeck PL, Reddy VS (2008). Structure of Seneca Valley Virus-001: an oncolytic picornavirus representing a new genus. Structure.

[159] Hales LM, Knowles NJ, Reddy PS, Xu L, Hay C, Hallenbeck PL (2008). Complete genome sequence analysis of Seneca Valley virus-001, a novel oncolytic picornavirus. J Gen Virol.

[160] Venkataraman S, Reddy SP, Loo J, Idamakanti N, Hallenbeck PL, Reddy VS (2008). Crystallization and preliminary X-ray diffraction studies of Seneca Valley virus-001, a new member of the Picornaviridae family. Acta Crystallogr Sect F Struct Biol Cryst Commun.

[161] Reddy PS, Burroughs KD, Hales LM, Ganesh S, Jones BH, Idamakanti N, Hay C, Li SS, Skele KL, Vasko AJ, Yang J, Watkins DN, Rudin CM, Hallenbeck PL (2007). Seneca Valley virus, a systemically deliverable oncolytic picornavirus, and the treatment of neuroendocrine cancers. J Natl Cancer Inst.

[162] Rudin CM, Poirier JT, Senzer NN, Stephenson J, Loesch D, Burroughs KD, Reddy PS, Hann CL, Hallenbeck PL (2011). Phase I clinical study of Seneca Valley Virus (SVV-001), a replication-competent picornavirus, in advanced solid tumors with neuroendocrine features. Clin Cancer Res.

[163] Obuchi M, Fernandez M, Barber GN (2003). Development of recombinant vesicular stomatitis viruses that exploit defects in host defense to augment specific oncolytic activity. J Virol.

[164] Saloura V, Wang LC, Fridlender ZG, Sun J, Cheng G, Kapoor V, Sterman DH, Harty RN, Okumura A, Barber GN, Vile RG, Federspiel MJ, Russell SJ (2010). Evaluation of an attenuated vesicular stomatitis virus vector expressing interferon-beta for use in malignant pleural mesothelioma: heterogeneity in interferon responsiveness defines potential efficacy. Hum Gene Ther.

[165] von Kobbe C, van Deursen JM, Rodrigues JP, Sitterlin D, Bachi A, Wu X, Wilm M, Carmo-Fonseca M, Izaurralde E (2000). Vesicular stomatitis virus matrix protein inhibits host cell gene expression by targeting the nucleoporin Nup98. Mol Cell.

[166] Wu L, Huang TG, Meseck M, Altomonte J, Ebert O, Shinozaki K, García-Sastre A, Fallon J, Mandeli J, Woo SL (2008). rVSV(M Delta 51)-M3 is an effective and safe oncolytic virus for cancer therapy. Hum Gene Ther.

[167] Jenks N, Myers R, Greiner SM, Thompson J, Mader EK, Greenslade A, Griesmann GE, Federspiel MJ, Rakela J, Borad MJ, Vile RG, Barber GN, Meier TR (2010). Safety studies on intrahepatic or intratumoral injection of oncolytic vesicular stomatitis virus expressing interferon-beta in rodents and nonhuman primates. Hum Gene Ther.

[168] Qiao J, Wang H, Kottke T, White C, Twigger K, Diaz RM, Thompson J, Selby P, de Bono J, Melcher A, Pandha H, Coffey M, Vile R, Harrington K (2008). Cyclophosphamide facilitates antitumor efficacy against subcutaneous tumors following intravenous delivery of reovirus. Clin Cancer Res.

[169] Kolb EA, Sampson V, Stabley D, Walter A, Sol-Church K, Cripe T, Hingorani P, Ahern CH, Weigel BJ, Zwiebel J, Blaney SM (2015). A phase I trial and viral clearance study of reovirus (Reolysin) in children with relapsed or refractory extra-cranial solid tumors: a Children's Oncology Group Phase I Consortium report. Pediatr Blood Cancer.

[170] Gujar SA, Clements D, Dielschneider R, Helson E, Marcato P, Lee PW (2014). Gemcitabine enhances the efficacy of reovirus-based oncotherapy through anti-tumour immunological mechanisms. Br J Cancer.

[171] Lolkema MP, Arkenau HT, Harrington K, Roxburgh P, Morrison R, Roulstone V, Twigger K, Coffey M, Mettinger K, Gill G, Evans TR, de Bono JS (2011). A phase I study of the combination of intravenous reovirus type 3 Dearing and gemcitabine in patients with advanced cancer. Clin Cancer Res.

[172] Ranki T, Pesonen S, Hemminki A, Partanen K, Kairemo K, Alanko T, Lundin J, Linder N, Turkki R, Ristimäki A, Jäger E, Karbach J, Wahle C (2016). Phase I study with ONCOS-102 for the treatment of solid tumors - an evaluation of clinical response and exploratory analyses of immune markers. J Immunother Cancer.

[173] Keir ME, Butte MJ, Freeman GJ, Sharpe AH (2008). PD-1 and its ligands in tolerance and immunity. Annu Rev Immunol.

[174] Rajani K, Parrish C, Kottke T, Thompson J, Zaidi S, Ilett L, Shim KG, Diaz RM, Pandha H, Harrington K, Coffey M, Melcher A, Vile R (2016). Combination Therapy With Reovirus and Anti-PD-1 Blockade Controls Tumor Growth Through Innate and Adaptive Immune Responses. Mol Ther.

[175] Woller N, Gürlevik E, Fleischmann-Mundt B, Schumacher A, Knocke S, Kloos AM, Saborowski M, Geffers R, Manns MP, Wirth TC, Kubicka S, Kühnel F (2015). Viral Infection of Tumors Overcomes Resistance to PD-1-immunotherapy by Broadening Neoantigenome-directed T-cell Responses. Mol Ther.

[176] Shen W, Patnaik MM, Ruiz A, Russell SJ, Peng KW (2016). Immunovirotherapy with vesicular stomatitis virus and PD-L1 blockade enhances therapeutic outcome in murine acute myeloid leukemia. Blood.

[177] Cockle JV, Rajani K, Zaidi S, Kottke T, Thompson J, Diaz RM, Shim K, Peterson T, Parney IF, Short S, Selby P, Ilett E, Melcher A, Vile R (2016). Combination viroimmunotherapy with checkpoint inhibition to treat glioma, based on location-specific tumor profiling. Neuro-oncol.

[178] Engeland CE, Grossardt C, Veinalde R, Bossow S, Lutz D, Kaufmann JK, Shevchenko I, Umansky V, Nettelbeck DM, Weichert W, Jäger D, von Kalle C, Ungerechts G (2014). CTLA-4 and PD-L1 checkpoint blockade enhances oncolytic measles virus therapy. Mol Ther.

[179] Minev B, Kohrt H, Kilinc M, Chen N, Feng A, Pessian M, Geissinger U, Haefner E, Tsoneva D, Bozhilov K, Sagiv-Barfi I, Zhao X, Rajesekaran N (2014). Combination immunotherapy with oncolytic vaccinia virus and checkpoint inhibitor following local tumor irradiation. Journal for Immunotherapy of Cancer.

[180] Barber DL, Wherry EJ, Masopust D, Zhu B, Allison JP, Sharpe AH, Freeman GJ, Ahmed R (2006). Restoring function in exhausted CD8 T cells during chronic viral infection. Nature.

[181] Liu J, Spurrel J, Shi ZQ, Chen W, Morris DG (2015). Abstract 5355: Oncolytic viral therapy with immune modulation is an effective novel treatment strategy for non-small cell lung cancer. Cancer Res.

[182] Roulstone V, Pedersen M, Kyula J, Mansfield D, Khan AA, McEntee G, Wilkinson M, Karapanagiotou E, Coffey M, Marais R, Jebar A, Errington-Mais F, Melcher A (2015). BRAF- and MEK-Targeted Small Molecule Inhibitors Exert Enhanced Antimelanoma Effects in Combination With Oncolytic Reovirus Through ER Stress. Mol Ther.

[183] Puzanov I, Milhem MM, Minor D, Hamid O, Li A, Chen L, Chastain M, Gorski KS, Anderson A, Chou J, Kaufman HL, Andtbacka RH (2016). Talimogene Laherparepvec in Combination With Ipilimumab in Previously Untreated, Unresectable Stage IIIB-IV Melanoma. J Clin Oncol.

[184] Park HJ, Park JS, Jeong YH, Son J, Ban YH, Lee BH, Chen L, Chang J, Chung DH, Choi I, Ha SJ (2015). PD-1 upregulated on regulatory T cells during chronic virus infection enhances the suppression of CD8+ T cell immune response via the interaction with PD-L1 expressed on CD8+ T cells. J Immunol.

[185] Puhlmann M, Brown CK, Gnant M, Huang J, Libutti SK, Alexander HR, Bartlett DL (2000). Vaccinia as a vector for tumor-directed gene therapy: biodistribution of a thymidine kinase-deleted mutant. Cancer Gene Ther.

[186] Hengstschläger M, Knöfler M, Müllner EW, Ogris E, Wintersberger E, Wawra E (1994). Different regulation of thymidine kinase during the cell cycle of normal versus DNA tumor virus-transformed cells. J Biol Chem.

[187] Buller RM, Chakrabarti S, Moss B, Fredrickson T (1988). Cell proliferative response to vaccinia virus is mediated by VGF. Virology.

[188] Zeh HJ, Downs-Canner S, McCart JA, Guo ZS, Rao UN, Ramalingam L, Thorne SH, Jones HL, Kalinski P, Wieckowski E, O’Malley ME, Daneshmand M, Hu K (2015). First-in-man study of western reserve strain oncolytic vaccinia virus: safety, systemic spread, and antitumor activity. Mol Ther.

[189] Kim JH, Oh JY, Park BH, Lee DE, Kim JS, Park HE, Roh MS, Je JE, Yoon JH, Thorne SH, Kirn D, Hwang TH (2006). Systemic armed oncolytic and immunologic therapy for cancer with JX-594, a targeted poxvirus expressing GM-CSF. Mol Ther.

[190] Zhang Q, Yu YA, Wang E, Chen N, Danner RL, Munson PJ, Marincola FM, Szalay AA (2007). Eradication of solid human breast tumors in nude mice with an intravenously injected light-emitting oncolytic vaccinia virus. Cancer Res.

[191] Krug LM, Zauderer MG, Adusumili PS, McGee E, Sepkowitz K, Klang M, Yu YA, Scigalla P, Rusch VW (2015). Phase I study of intra-pleural administration of GL-ONC1, an oncolytic vaccinia virus, in patients with malignant pleural effusion. ASCO Meeting Abstracts.

[192] Lauer U, Zimmermann M, Sturm J, Koppenhoefer U, Bitzer M, Malek NP, Glatzle J, Koenigsrainer A, Moehle R, Fend F, Pfannenberg C, Auth T, Yu T (2013). Phase I/II clinical trial of a genetically modified and oncolytic vaccinia virus GL-ONC1 in patients with unresactable, chemotherapy-resistant peritoneal carcinomatosis. J Clin Oncol.

[193] Jaime JC, Young AM, Mateo J, Yap TA, Denholm KA, Shah KJ, Tunariu N, Sassi S, Karapanegiotou L, Mansfield D, Molife LR, Harrington KJ, De Bono JS (2012). Phase I clinical trial of a genetically modified and oncolytic vaccinia virus GL-ONC1 with green fluorescent protein imaging. J Clin Oncol.

[194] Khan KH, Young AM, Mateo J, Tunariu N, Yap TA, Tan DS, Mansfield D, Wong M, Riisnaes R, Harrington KJ, De Bono JS (2013). Phase I clinical trial of a genetically modified and oncolytic vaccinia virus GL-ONC1 with green fluorescent protein imaging (NCT009794131). J Clin Oncol.

[195] Fueyo J, Gomez-Manzano C, Alemany R, Lee PS, McDonnell TJ, Mitlianga P, Shi YX, Levin VA, Yung WK, Kyritsis AP (2000). A mutant oncolytic adenovirus targeting the Rb pathway produces anti-glioma effect in vivo. Oncogene.

[196] Kim KH, Dmitriev IP, Saddekni S, Kashentseva EA, Harris RD, Aurigemma R, Bae S, Singh KP, Siegal GP, Curiel DT, Alvarez RD (2013). A phase I clinical trial of Ad5/3-Δ24, a novel serotype-chimeric, infectivity-enhanced, conditionally-replicative adenovirus (CRAd), in patients with recurrent ovarian cancer. Gynecol Oncol.

[197] Pesonen S, Diaconu I, Cerullo V, Escutenaire S, Raki M, Kangasniemi L, Nokisalmi P, Dotti G, Guse K, Laasonen L, Partanen K, Karli E, Haavisto E (2012). Integrin targeted oncolytic adenoviruses Ad5-D24-RGD and Ad5-RGD-D24-GMCSF for treatment of patients with advanced chemotherapy refractory solid tumors. Int J Cancer.

[198] Bramante S, Koski A, Kipar A, Diaconu I, Liikanen I, Hemminki O, Vassilev L, Parviainen S, Cerullo V, Pesonen SK, Oksanen M, Heiskanen R, Rouvinen-Lagerström N (2014). Serotype chimeric oncolytic adenovirus coding for GM-CSF for treatment of sarcoma in rodents and humans. Int J Cancer.

[199] Lang FF, Conrad C, Gomez-Manzano C, Tufaro F, Sawaya R, Weinberg J, Prabhu S, Fuller G, Aldape K, Fueyo J (2014). Phase I clinical trial of oncolytic virus delta-24-rgd (DNX-2401) with biological endpoints: implications for viro-immunotherapy. Neuro-oncol.

[200] Holterman L, Vogels R, van der Vlugt R, Sieuwerts M, Grimbergen J, Kaspers J, Geelen E, van der Helm E, Lemckert A, Gillissen G, Verhaagh S, Custers J, Zuijdgeest D (2004). Novel replication-incompetent vector derived from adenovirus type 11 (Ad11) for vaccination and gene therapy: low seroprevalence and non-cross-reactivity with Ad5. J Virol.

[201] Kuhn I, Harden P, Bauzon M, Chartier C, Nye J, Thorne S, Reid T, Ni S, Lieber A, Fisher K, Seymour L, Rubanyi GM, Harkins RN, Hermiston TW (2008). Directed evolution generates a novel oncolytic virus for the treatment of colon cancer. PLoS One.

[202] Garcia-Carbonero R, Gil-Martin M, Calvo E, Prados S, De la Portilla F, Salazar R, Santos C, Sanchez-Gastaldo A, Duran H, Sanjuan X, Bozada JM, Boni V, Jurado M (2014). A phase 1 mechanism of action study of intratumoral or intravenous administration of enadenotucirev, an oncolytic Ad11/Ad3 chimeric group B adenovirus in colon cancer patients undergoing resection of primary tumor. ASCO Meeting Abstracts.

[203] Rodriguez R, Schuur ER, Lim HY, Henderson GA, Simons JW, Henderson DR (1997). Prostate attenuated replication competent adenovirus (ARCA) CN706: a selective cytotoxic for prostate-specific antigen-positive prostate cancer cells. Cancer Res.

[204] Yu DC, Sakamoto GT, Henderson DR (1999). Identification of the transcriptional regulatory sequences of human kallikrein 2 and their use in the construction of calydon virus 764, an attenuated replication competent adenovirus for prostate cancer therapy. Cancer Res.

[205] Fujiwara T, Shirakawa Y, Kagawa S (2011). Telomerase-specific oncolytic virotherapy for human gastrointestinal cancer. Expert Rev Anticancer Ther.

[206] Kojima T, Watanabe Y, Hashimoto Y, Kuroda S, Yamasaki Y, Yano S, Ouchi M, Tazawa H, Uno F, Kagawa S, Kyo S, Mizuguchi H, Urata Y (2010). In vivo biological purging for lymph node metastasis of human colorectal cancer by telomerase-specific oncolytic virotherapy. Ann Surg.

[207] Moehler MH, Zeidler M, Wilsberg V, Cornelis JJ, Woelfel T, Rommelaere J, Galle PR, Heike M (2005). Parvovirus H-1-induced tumor cell death enhances human immune response in vitro via increased phagocytosis, maturation, and cross-presentation by dendritic cells. Hum Gene Ther.

[208] Geletneky K, Kiprianova I, Ayache A, Koch R, Herrero Y Calle M, Deleu L, Sommer C, Thomas N, Rommelaere J, Schlehofer JR (2010). Regression of advanced rat and human gliomas by local or systemic treatment with oncolytic parvovirus H-1 in rat models. Neuro-oncol.

[209] Raykov Z, Grekova S, Galabov AS, Balboni G, Koch U, Aprahamian M, Rommelaere J (2007). Combined oncolytic and vaccination activities of parvovirus H-1 in a metastatic tumor model. Oncol Rep.

[210] Todd D (2000). Circoviruses: immunosuppressive threats to avian species: a review. Avian Pathol.

[211] Noteborn MH (2004). Chicken anemia virus induced apoptosis: underlying molecular mechanisms. Vet Microbiol.

[212] Danen-van Oorschot AA, Voskamp P, Seelen MC, van Miltenburg MH, Bolk MW, Tait SW, Boesen-de Cock JG, Rohn JL, Borst J, Noteborn MH (2004). Human death effector domain-associated factor interacts with the viral apoptosis agonist Apoptin and exerts tumor-preferential cell killing. Cell Death Differ.

[213] Maddika S, Booy EP, Johar D, Gibson SB, Ghavami S, Los M (2005). Cancer-specific toxicity of apoptin is independent of death receptors but involves the loss of mitochondrial membrane potential and the release of mitochondrial cell-death mediators by a Nur77-dependent pathway. J Cell Sci.

[214] Zhuang SM, Shvarts A, van Ormondt H, Jochemsen AG, van der Eb AJ, Noteborn MH (1995). Apoptin, a protein derived from chicken anemia virus, induces p53-independent apoptosis in human osteosarcoma cells. Cancer Res.

[215] Peng Y (2012). Potential prognostic tumor biomarkers in triple-negative breast carcinoma. Beijing Da Xue Xue Bao.

[216] Olijslagers SJ, Zhang YH, Backendorf C, Noteborn MH (2007). Additive cytotoxic effect of apoptin and chemotherapeutic agents paclitaxel and etoposide on human tumour cells. Basic Clin Pharmacol Toxicol.

[217] Natesan S, Kataria JM, Dhama K, Bhardwaj N, Sylvester A (2006). Anti-neoplastic effect of chicken anemia virus VP3 protein (apoptin) in Rous sarcoma virus-induced tumours in chicken. J Gen Virol.

[218] Connolly JL, Rodgers SE, Clarke P, Ballard DW, Kerr LD, Tyler KL, Dermody TS (2000). Reovirus-induced apoptosis requires activation of transcription factor NF-kappaB. J Virol.

[219] Clarke P, Meintzer SM, Wang Y, Moffitt LA, Richardson-Burns SM, Johnson GL, Tyler KL (2004). JNK regulates the release of proapoptotic mitochondrial factors in reovirus-infected cells. J Virol.

[220] Gollamudi R, Ghalib MH, Desai KK, Chaudhary I, Wong B, Einstein M, Coffey M, Gill GM, Mettinger K, Mariadason JM, Mani S, Goel S (2010). Intravenous administration of Reolysin, a live replication competent RNA virus is safe in patients with advanced solid tumors. Invest New Drugs.

[221] Vidal L, Pandha HS, Yap TA, White CL, Twigger K, Vile RG, Melcher A, Coffey M, Harrington KJ, DeBono JS (2008). A phase I study of intravenous oncolytic reovirus type 3 Dearing in patients with advanced cancer. Clin Cancer Res.

[222] Forsyth P, Roldán G, George D, Wallace C, Palmer CA, Morris D, Cairncross G, Matthews MV, Markert J, Gillespie Y, Coffey M, Thompson B, Hamilton M (2008). A phase I trial of intratumoral administration of reovirus in patients with histologically confirmed recurrent malignant gliomas. Mol Ther.

[223] Morris DG, Feng X, DiFrancesco LM, Fonseca K, Forsyth PA, Paterson AH, Coffey MC, Thompson B (2013). REO-001: A phase I trial of percutaneous intralesional administration of reovirus type 3 dearing (Reolysin®) in patients with advanced solid tumors. Invest New Drugs.

[224] Kicielinski KP, Chiocca EA, Yu JS, Gill GM, Coffey M, Markert JM (2014). Phase 1 clinical trial of intratumoral reovirus infusion for the treatment of recurrent malignant gliomas in adults. Mol Ther.

[225] Comins C, Spicer J, Protheroe A, Roulstone V, Twigger K, White CM, Vile R, Melcher A, Coffey MC, Mettinger KL, Nuovo G, Cohn DE, Phelps M (2010). REO-10: a phase I study of intravenous reovirus and docetaxel in patients with advanced cancer. Clin Cancer Res.

[226] Karapanagiotou EM, Roulstone V, Twigger K, Ball M, Tanay M, Nutting C, Newbold K, Gore ME, Larkin J, Syrigos KN, Coffey M, Thompson B, Mettinger K (2012). Phase I/II trial of carboplatin and paclitaxel chemotherapy in combination with intravenous oncolytic reovirus in patients with advanced malignancies. Clin Cancer Res.

[227] Ocean AJ, Bekaii-Saab TS, Chaudhary I, Palmer R, Christos PJ, Mercado A, Florendo EO, Rosales VA, Ruggiero JT, Popa EC, Wilson M, Ghalib MH, Hou Y (2013). A multicenter phase I study of intravenous administration of reolysin in combination with irinotecan/fluorouracil/leucovorin (FOLFIRI) in patients (pts) with oxaliplatin-refractory/intolerant KRAS-mutant metastatic colorectal cancer (mCRC). ASCO Meeting Abstracts.

[228] Skelding KA, Barry RD, Shafren DR (2012). Enhanced oncolysis mediated by Coxsackievirus A21 in combination with doxorubicin hydrochloride. Invest New Drugs.

[229] Allen C, Paraskevakou G, Iankov I, Giannini C, Schroeder M, Sarkaria J, Puri RK, Russell SJ, Galanis E (2008). Interleukin-13 displaying retargeted oncolytic measles virus strains have significant activity against gliomas with improved specificity. Mol Ther.

[230] Peng KW, Facteau S, Wegman T, O’Kane D, Russell SJ (2002). Non-invasive in vivo monitoring of trackable viruses expressing soluble marker peptides. Nat Med.

[231] Dingli D, Peng KW, Harvey ME, Greipp PR, O’Connor MK, Cattaneo R, Morris JC, Russell SJ (2004). Image-guided radiovirotherapy for multiple myeloma using a recombinant measles virus expressing the thyroidal sodium iodide symporter. Blood.

[232] Msaouel P, Iankov ID, Allen C, Aderca I, Federspiel MJ, Tindall DJ, Morris JC, Koutsilieris M, Russell SJ, Galanis E (2009). Noninvasive imaging and radiovirotherapy of prostate cancer using an oncolytic measles virus expressing the sodium iodide symporter. Mol Ther.

[233] Myers R, Harvey M, Kaufmann TJ, Greiner SM, Krempski JW, Raffel C, Shelton SE, Soeffker D, Zollman P, Federspiel MJ, Blanco M, Galanis E (2008). Toxicology study of repeat intracerebral administration of a measles virus derivative producing carcinoembryonic antigen in rhesus macaques in support of a phase I/II clinical trial for patients with recurrent gliomas. Hum Gene Ther.

[234] Myers RM, Greiner SM, Harvey ME, Griesmann G, Kuffel MJ, Buhrow SA, Reid JM, Federspiel M, Ames MM, Dingli D, Schweikart K, Welch A, Dispenzieri A (2007). Preclinical pharmacology and toxicology of intravenous MV-NIS, an oncolytic measles virus administered with or without cyclophosphamide. Clin Pharmacol Ther.

[235] Peng KW, Frenzke M, Myers R, Soeffker D, Harvey M, Greiner S, Galanis E, Cattaneo R, Federspiel MJ, Russell SJ (2003). Biodistribution of oncolytic measles virus after intraperitoneal administration into Ifnar-CD46Ge transgenic mice. Hum Gene Ther.

[236] Schirrmacher V, Ahlert T, Pröbstle T, Steiner HH, Herold-Mende C, Gerhards R, Hagmüller E, Steiner HH (1998). Immunization with virus-modified tumor cells. Semin Oncol.

[237] Schirrmacher V, Bai L, Umansky V, Yu L, Xing Y, Qian Z (2000). Newcastle disease virus activates macrophages for anti-tumor activity. Int J Oncol.

[238] Termeer CC, Schirrmacher V, Bröcker EB, Becker JC (2000). Newcastle disease virus infection induces B7-1/B7-2-independent T-cell costimulatory activity in human melanoma cells. Cancer Gene Ther.

[239] Lam KM (1996). Growth of Newcastle disease virus in chicken macrophages. J Comp Pathol.

[240] Ravindra PV, Tiwari AK, Ratta B, Chaturvedi U, Palia SK, Chauhan RS (2009). Newcastle disease virus-induced cytopathic effect in infected cells is caused by apoptosis. Virus Res.

[241] Ravindra PV, Tiwari AK, Ratta B, Bais MV, Chaturvedi U, Palia SK, Sharma B, Chauhan RS (2009). Time course of Newcastle disease virus-induced apoptotic pathways. Virus Res.

[242] Roberts S, Buasen P, Incao B, Groene S, Duhon C, McDaniel G, Welch A, Leitner T, Miller JA, Rabin H, Lorence R (2001). PV701, a naturally attenuated strain of Newcastle disease virus, has a broad spectrum of oncolytic activity against human tumor xenografts. Proc Am Assoc Cancer Res.

[243] Bergelson JM, Shepley MP, Chan BM, Hemler ME, Finberg RW (1992). Identification of the integrin VLA-2 as a receptor for echovirus 1. Science.

[244] Shafren DR, Sylvester D, Johansson ES, Campbell IG, Barry RD (2005). Oncolysis of human ovarian cancers by echovirus type 1. Int J Cancer.

[245] Berry LJ, Au GG, Barry RD, Shafren DR (2008). Potent oncolytic activity of human enteroviruses against human prostate cancer. Prostate.

[246] Doniņa S, Strēle I Proboka G, Auziņš J, Alberts P, Jonsson B, Venskus D, Muceniece A (2015). Adapted ECHO-7 virus Rigvir immunotherapy (oncolytic virotherapy) prolongs survival in melanoma patients after surgical excision of the tumour in a retrospective study. Melanoma Res.

